# Gonadal white adipose tissue-derived exosomal MiR-222 promotes obesity-associated insulin resistance

**DOI:** 10.18632/aging.103891

**Published:** 2020-11-10

**Authors:** Dameng Li, Huichen Song, Linghu Shuo, Lei Wang, Ping Xie, Weili Li, Jiachen Liu, Yafei Tong, Chen-Yu Zhang, Xiaohong Jiang, Jing Li, Yujing Zhang

**Affiliations:** 1 Nanjing DrumTower Hospital Center of Molecular Diagnostic and Therapy, State Key Laboratory of Pharmaceutical Biotechnology, Jiangsu Engineering Research Center for MicroRNA Biology and Biotechnology, NJU Advanced Institute of Life Sciences (NAILS), NJU Institute of AI Biomedicine and Biotechnology, School of Life Sciences, Nanjing University, Nanjing 210023, China

**Keywords:** miR-222, adipose, insulin resistance, liver, skeletal muscle

## Abstract

In this study, we investigated the role of serum exosomal miR-222 in obesity-related insulin resistance. Bioinformatics analyses showed that miR-222 levels were significantly upregulated in the white adipose tissue of obese patients with insulin resistance (GSE25402 dataset) and in serum samples from type 2 diabetes mellitus (T2DM) patients (GSE90028 dataset). Moreover, analysis of miRNA expression in adipose tissue-specific Dicer knockout mice (GitHub dataset) and diabetic model mice (GSE81976 and GSE85101 datasets), gonadal white adipose tissue (gWAT) was the main source of serum exosomal miR-222. MiR-222 levels were significantly elevated in the serum, serum exosomes and gWAT of mice fed a high-fat diet (HFD), and there was a corresponding downregulation of IRS1 and phospho-AKT levels in their liver and skeletal muscle tissues, which correlated with impaired insulin sensitivity and glucose intolerance. These effects were abrogated by surgically removing the gWAT from the HFD-fed mice. Thus, gWAT-derived serum exosomal miR-222 appears to promote insulin resistance in the liver and skeletal muscle of HFD-fed obese mice by suppressing IRS1 expression.

## INTRODUCTION

Diabetes mellitus is a heterogeneous metabolic disorder and a leading cause of deaths worldwide [1, 2]. Type 2 diabetes mellitus (T2DM) is characterized by insulin resistance and accounts for more than 90% of diabetes cases [3]. Obesity is a common etiological factor that contributes to insulin resistance [4]. The rapid rise in the incidence of obesity worldwide has also resulted in a parallel increase in T2DM cases. Obesity is characterized by excessive accumulation and storage of body fat. The adipose tissue plays a major role in the pathogenesis of obesity-related insulin resistance by secreting several pro-inflammatory adipokines that regulate obesity-induced insulin resistance [4–10]. However, therapies that target these factors have only limited benefits, thereby suggesting the involvement of other mechanisms in obesity-induced insulin resistance. Several studies have shown that exosomal miRNAs act as novel adipokines and regulate insulin biogenesis and function [11–17].

MicroRNAs are small noncoding RNAs that are 19 to 24 nucleotides in length. They repress gene expression by binding to the 3'-UTR of their specific target mRNAs and promote translational inhibition and degradation of their target mRNAs by acting in concert with the RNA-induced silencing complex or RISC [18–24]. Moreover, in addition to their abundant expression in the cells and tissues, miRNAs have also been detected in the circulation system [25–27]. Various circulating miRNAs are found in the exosomes, which are 50–200 nm vesicles that are released from donor cells and contain cargo that includes miRNAs, mRNAs, and proteins, which regulate the function of the recipient cells in the immediate vicinity or in distant tissues [28–30]. The exosomal miRNAs regulate various biological processes, including development, immune responses, and tumorigenesis [31–35]. Adipose tissue is an important source of circulating exosomal miRNAs [14]. Several reports suggest that the circulating adipose tissue-derived exosomal miRNAs modulate insulin sensitivity [11–17]. Adipocyte-derived miR-27a induces insulin resistance by repressing peroxisome proliferator-activated receptor γ or PPARγ [12]. Adipose tissue macrophages secrete miR-155, which inhibits insulin signaling by suppressing PPARγ [16]. Furthermore, adipose tissues secrete miR-99b, which inhibits the expression of fibroblast growth factor 21 (FGF21) in the liver, thereby altering the metabolism in multiple tissues [14].

In this study, we analyzed the mechanisms through which serum exosomal miR-222 regulates obesity-related insulin resistance.

## RESULTS

### MiR-222 is significantly elevated in both serum samples from T2DM patients and white adipose tissue samples from obese insulin resistance patients

**Figure 1:**
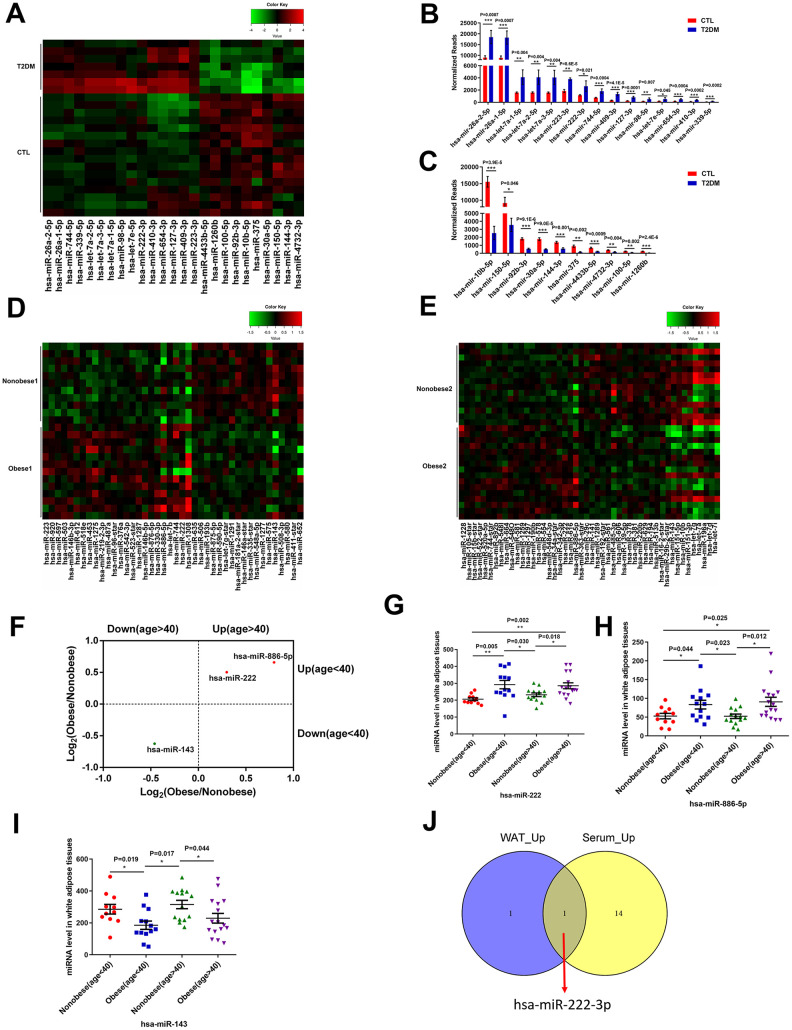
**MiR-222 expression is significantly increased in the serum of T2DM patients and the white adipose tissues of obese insulin-resistant patients.** (**A**) Heat map shows the differential expression of 25 miRNAs in the serum samples from T2DM patients (T2DM, n=7) and healthy subjects (CTL, n=16) using *P* < 0.05, FC (T2DM/CTL) > 2 or < 0.5, and at least 100 reads in all samples as the cut-off criteria. The color-coded key denotes the fold-changes. (**B**, **C**) The bar graphs show the normalized reads of the 15 upregulated and the 10 downregulated miRNAs in the serum of T2DM patients compared to the serum of healthy subjects. (**D**) The heat map shows the differential expression of 42 miRNAs in the WAT samples from the young non-obese group (Non-obese 1; n=11) and the young obese group (Obese 1; n=13) using *P* < 0.05 as the selection criterion. The color-coded key denotes the fold changes. (**E**) The heat map shows the differential expression of 49 miRNAs in the WAT samples from the old non-obese group (Non-obese 2; n=14) and the old obese group (Obese 2; n=16) using *P* < 0.05 as the selection criterion. The color-coded key denotes the fold changes. (**F**) The scatter plot shows the 3 miRNAs, miR-222, miR-143, and miR-886-5p that are differentially regulated consistently in the old and young obese individuals compared to the corresponding non-obese individuals. (**G**–**I**) The dot plots show the levels of (**G**) miR-222, (**H**) miR-886-5p, and (**I**) miR-143 in the WAT samples from the young non-obese, young obese, old non-obese, and old obese groups. (**J**) The Venn diagram shows that miR-222 is the only miRNA that is upregulated in both the T2DM patient serum (15 upregulated miRNAs) and obese (young and old) WAT (2 upregulated miRNAs) samples. Note: The results in (**A**–**C**) are based on the GSE90028 dataset. The results in (**D**–**I**) are based on the GSE25402 dataset. Note: T2DM, type 2 diabetes mellitus; WAT, white adipose tissue; Data are presented as the means ± SE; ^*^ *P* < 0.05, ^**^ *P* < 0.01, ^***^ *P* < 0.001.

We identified 291 mature miRNAs with at least 20 reads in the serum of T2DM patients (n=7) and healthy subjects (n=16) by analyzing the GSE90028 dataset from the NCBI GEO database (Supplementary Table 1). Among these, 152 serum miRNAs were differentially expressed miRNAs (103 upregulated and 49 downregulated) in the T2DM patients compared to the healthy subjects (Supplementary Figure 1). Furthermore, using a criteria of at least 100 reads in all samples, we identified 15 miRNAs with a fold change (FC) > 2 and 10 miRNAs with a FC < 0.5 in T2DM patients compared to healthy subjects (Figure 1A–1C).

Then, we analyzed the differentially expressed miRNAs in the white adipose tissues (WAT) from obese patients with insulin resistance compared to those from healthy subjects using the GSE25402 dataset. The patients were grouped into young non-obese (Non-obese 1; n=11), young obese (Obese 1; n=13), old non-obese (Non-obese 2; n=14) and old obese (Obese 2; n=16) groups (Supplementary Table 2). The WAT tissues of young obese patients showed 24 upregulated and 18 downregulated miRNAs compared to the young lean subjects using *P* < 0.05 as the cut-off criteria (Figure 1D; Supplementary Figure 2). The WAT tissues of older obese patients showed 22 upregulated and 26 downregulated miRNAs compared to the older lean subjects using *P* < 0.05 as the cut-off (Figure 1E, Supplementary Figure 2). The commonly differentially expressed miRNAs in both young and old obese patients were miR-222, miR-886-5p, and miR-143 (Figure 1F). In both young and old obese patients, miR-222 and miR-886-5p were upregulated (Figure 1G, 1H), and miR-143 was downregulated (Figure 1I).

We compared the differentially expressed miRNAs in the serum samples from the T2DM patients and WAT samples from the young and older obese patients and found that miR-222 was significantly upregulated in the serum samples from T2DM patients and the WAT samples from obese patients (Figure 1J).

### Gonadal white adipose tissue is a major source of serum exosomal miR-222

**Figure 2:**
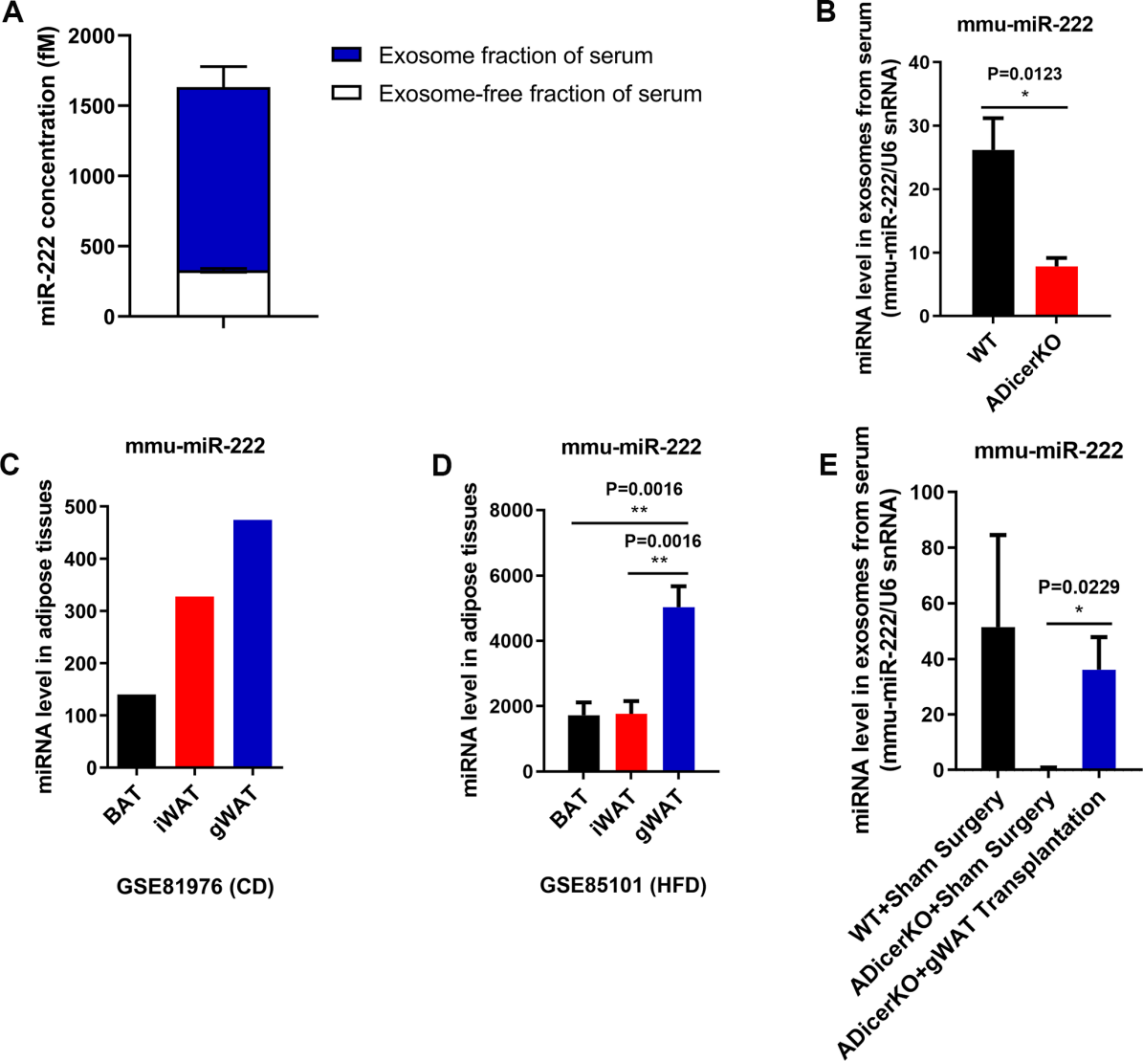
**Gonadal white adipose tissue is a major source of serum exosomal miR-222.** (**A**) QRT-PCR analysis shows the miR-222 levels in the exosome (n=5; blue portion of the bar) and exosome-free (n=5; white portion of the bar) fractions of the wild-type mouse serum. (**B**) The data from the GitHub database shows the miR-222 levels in the serum exosomes from the wild-type (black bar) and ADicerKO (adipose-tissue-specific knockout of Dicer; red bar) mice (n=4/group). (**C**) The GSE81976 dataset analysis shows the miR-222 levels in the brown adipose tissue (BAT; black bar; n=1), inguinal white adipose tissue (iWAT; red bar; n=1) and the gonadal white adipose tissue (gWAT; blue bar; n=1) of chow-fed mice. (**D**) The GSE85101 dataset analysis shows the miR-222 levels in the brown adipose tissue (black bar; n=3), inguinal white adipose tissue (red bar; n=3) and gonadal white adipose tissue (blue bar; n=3) from the HFD-fed mice. (**E**) The GitHub database analysis shows the levels of miR-222 in the serum exosomes from wild-type mice after sham surgery (n=3), ADicerKO mice after sham surgery (n=4), and ADicerKO mice after transplantation with wild-type-derived gonadal WAT (n=4). Note: The data are presented as the means ± SE; * *P* < 0.05, ** *P* < 0.01, *** *P* < 0.001.

QRT-PCR analysis showed that exosomal miR-222 levels were significantly higher than non-exosomal miR-222 in the mouse serum (Figure 2A). Furthermore, GitHub database analysis showed that miR-222 levels were significantly reduced in the serum exosomes from the adipose-tissue-specific Dicer knockout (ADicerKO) mice compared to those from the wild-type mice (Figure 2B). This suggests that most of the serum exosomal miR-222 is derived from the adipose tissues.

Adipose tissue sources include brown adipose tissue (BAT) and white adipose tissue (WAT). Moreover, the major sources of WAT are the inguinal WAT (iWAT) and gonadal WAT (gWAT). We analyzed miR-222 levels in the iWAT, gWAT, and BAT from wild-type and HFD-induced obese mice using the GSE81976 and GSE85101 datasets and found that despite differences in the miR-222 expression between wild-type and obese model mice, miR-222 levels were higher in the gWAT than in other adipose tissues (Figure 2C, 2D). Moreover, miR-222 levels were significantly higher in the gWAT from various obese mouse models compared to the controls (Supplementary Figure 3).

We then analyzed if gWAT was the major source of serum exosomal miR-222. GitHub database analysis showed that serum exosomal miR-222 levels from the ADicerKO mice that underwent sham surgery were significantly reduced compared to those from wild-type mice (Figure 2E). However, serum exosomal miR-222 levels were restored to 70% of normal in the ADicerKO mice after receiving gWAT transplants (Figure 2E). These data suggest that serum exosomal miR-222 is mostly derived from the gWAT.

### The elevated miR-222 in the serum and serum exosomes of high-fat diet (HFD)-induced obese mice are mainly derived from gonadal adipose tissues

Next, we used the high-fat diet (HFD)-fed mouse model of obesity-induced insulin resistance to confirm our findings. We fed male and female C57BL/6 mice with a high-fat diet (HFD) or a normal chow diet (CD) for 8 weeks. In 8 weeks, the body mass weight gain of the HFD-fed male mice was 68% compared to 18% for the CD-fed mice (Supplementary Figure 4A). Concurrently, the body mass weight gain of the HFD-fed female mice was 36% compared to 11% for the CD-fed female mice in 8 weeks (Supplementary Figure 5A). Both male and female HFD-fed mice showed impaired glucose tolerance and insulin resistance compared to the CD-fed mice (Supplementary Figures 4B, 4C, 5B, 5C). The impairments were more pronounced in the male HFD-fed mice compared to the female HFD-fed mice.

**Figure 3:**
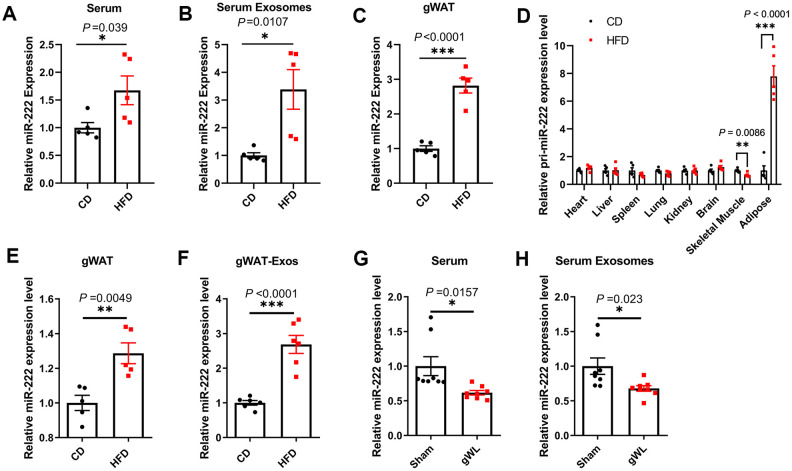
**Gonadal adipose tissues are the major source of the elevated miR-222 in the serum and serum exosomes of high-fat diet (HFD)-induced obese model mice.** (**A**–**C**) QRT-PCR analysis shows the relative miR-222 levels in the (**A**) serum, (**B**) serum exosomes, and (**C**) gonadal white adipose tissues (gWAT) harvested from the male CD-fed or HFD-fed mice (n=5 per group). (**D**) QRT-PCR analysis shows the relative levels of pri-miR-222 in the heart, liver, spleen, lung, kidney, brain, skeletal muscle and gonadal adipose tissue harvested from the male CD-fed or HFD-fed mice (n=5 per group). (**E**, **F**) QRT-PCR analysis shows the relative expression of miR-222 in the (**E**) *in-vitro* cultured gWAT tissue slices from CD-fed and HFD-fed mice (n=5 per group) and (**F**) exosomes from the culture medium of the *in vitro* cultured gWAT tissue slices from the CD-fed and HFD-fed mice (n=6 per group). The samples were extracted after *in vitro* culturing for 48h. (**G**, **H**) The relative levels of miR-222 in the (**G**) serum and (**H**) serum exosomes from gWAT-lipectomized (gWL) and sham-operated (Sham) HFD mice (n=8 per group). Note: The data are presented as the means ± SE. * *P* < 0.05, ** *P* < 0.01, *** *P* < 0.001.

We then detected miR-222 levels in the serum, serum exosomes and gWAT of HFD- and the CD-fed mice. The miR-222 levels were significantly higher in the serum (Figure 3A and Supplementary Figure 5D), serum exosomes (Figure 3B and Supplementary Figure 5E) and gWAT (Figure 3C and Supplementary Figure 5F) of the HFD-induced obese male and female mice compared to the CD-fed male and female mice. We chose male mice for further experiments since there were no significant differences of miR-222 levels between the male and female HFD-fed mice.

To identify the source of elevated miR-222 in the serum and the serum exosomes of HFD mice, we first analyzed the level of pri-miR-222 expression in the heart, liver, spleen, lung, kidney, brain, skeletal muscle and adipose tissues by QRT-PCR using a pri-miR-222-specific Taqman probe. The levels of pri-miR-222 was significantly higher only in the gWAT from HFD-fed mice compared to the CD-fed mice, whereas, the levels of pri-miR-222 were comparable in other tissues for both HFD- and CD-fed mice (Figure 3D). These results suggest that gWAT is the most likely the source of elevated serum exosomal miR-222 in the HFD mice.

We then surgically harvested gWAT from the CD- and HFD-fed mice and cultured them *in vitro*. After 48 h of culture, we analyzed the levels of miR-222 in the gWAT tissue slices and the exosomes in the culture medium. The miR-222 levels were significantly higher in the cultured gWAT tissue slices and the gWAT-derived exosomes from HFD-fed mice compared to those from the CD-fed mice (Figure 3E, 3F). Next, we surgically removed the gWAT from the HFD mice at 8 weeks after HFD-diet and measured the levels of miR-222 in the serum and serum exosomes of gWAT-lipectomized (gWL) and sham-operated (sham) HFD-fed mice after 1 week. The miR-222 levels were significantly lower in the serum and serum exosomes of the gWL-HFD mice compared to those in the sham-HFD mice (Figure 3G, 3H). Taken together, these results demonstrate that gWAT is a major source of increased miR-222 levels in the serum and serum exosomes of HFD-fed obese model mice.

### Liver and skeletal muscle are the major target tissues of gWAT-derived exosomal miR-222

**Figure 4:**
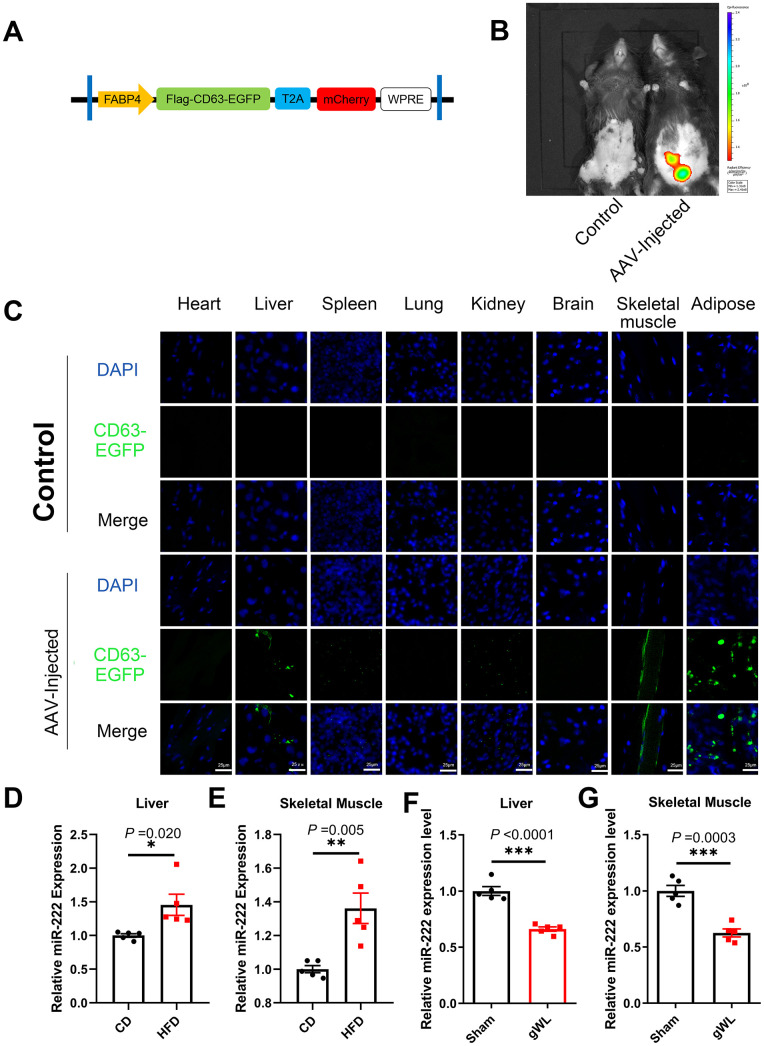
**Liver and skeletal muscle are the major target tissues of gWAT-derived exosomal miR-222.** (**A**) Diagrammatic representation of the AAV plasmid vector construct, HBAAV2/9-FABP4-3xflag-CD63-EGFP-T2A-mCherry. (**B**) Representative images show the *in vivo* imaging results in control (left) and AAV-injected (right) mice. The distribution of mCherry was detected. The expression of mCherry was observed in the gWAT of the AAV-injected mouse but was absent in the control. (**C**) Representative confocal microscopic images show the tissue distribution of the gWAT-derived exosomes that are marked by CD63-EGFP (green). The nuclei are stained using DAPI (blue). (**D**, **E**) QRT-PCR analysis results show the relative expression of miR-222 in the (**D**) liver and (**E**) skeletal muscle tissues from the CD-fed and HFD-fed mice (n=5 per group). (**F**, **G**) QRT-PCR analysis results show the relative expression of miR-222 in the (**F**) liver and (**G**) skeletal muscle tissues from gWAT-lipectomized (gWL) and sham-operated (Sham) HFD mice (n=5 per group). The data are presented as the means ± SE. * *P* < 0.05, ** *P* < 0.01, *** *P* < 0.001.

Next, we analyzed the target tissues of gWAT-derived exosomal miR-222 by constructing an adeno-associated vector (AAV) expressing a fusion protein CD63-EGFP and an indicator protein mCherry under a FABP4 promoter (Figure 4A). We injected the AAV viruses *in situ* into the gWAT of HFD-fed mice at 8 weeks after HFD-diet. At 8 weeks after injection, *in vivo* imaging results showed that mCherry was expressed only in the gWAT of AAV-injected mice (Figure 4B). Therefore, gWAT was the only source tissue that produced EGFP-labeled exosomes. Then, at 8 weeks after injection, we performed confocal microscopy and observed EGFP fluorescence in the liver, spleen, kidney, skeletal muscle and gWAT of the AAV-injected mice (Figure 4C). These results suggest that gWAT-derived exosomes are transported to the liver, spleen, kidney and skeletal muscle tissues. Moreover, EGFP fluorescence was significantly higher in the liver and skeletal muscle tissues, which are also the major target tissues for insulin to maintain glucose homeostasis. Therefore, we focused on the liver and skeletal muscle tissues in our further experiments.

QRT-PCR results showed that miR-222 levels were significantly higher in the liver and skeletal muscle tissues of the HFD-fed male mice compared to those from the CD-fed male mice (Figure 4D, 4E). Similar trends were also observed in HFD-fed female mice (Supplementary Figure 6A, 6B). However, as shown in Figure 3D, the pri-miR-222 levels were similar in the liver and skeletal muscle tissues of the HFD-fed and CD-fed mice. This suggests that the elevated miR-222 levels in the liver and skeletal muscle tissues of HFD mice are probably derived from an increase in the gWAT-derived exosomal miR-222. To further confirm this hypothesis, we analyzed miR-222 levels in the liver and skeletal muscle tissues of the gWAT-lipectomized (gWL) and Sham mice at 1 week after surgery. The miR-222 levels were significantly lower in the liver and skeletal muscle tissues of the gWL mice compared to those from the Sham mice (Figure 4F, 4G). This confirms that the elevated miR-222 levels in the liver and skeletal muscle tissues of the HFD-fed mice were derived from the gWAT exosomes.

### IRS1 is a direct target of miR-222

**Figure 5:**
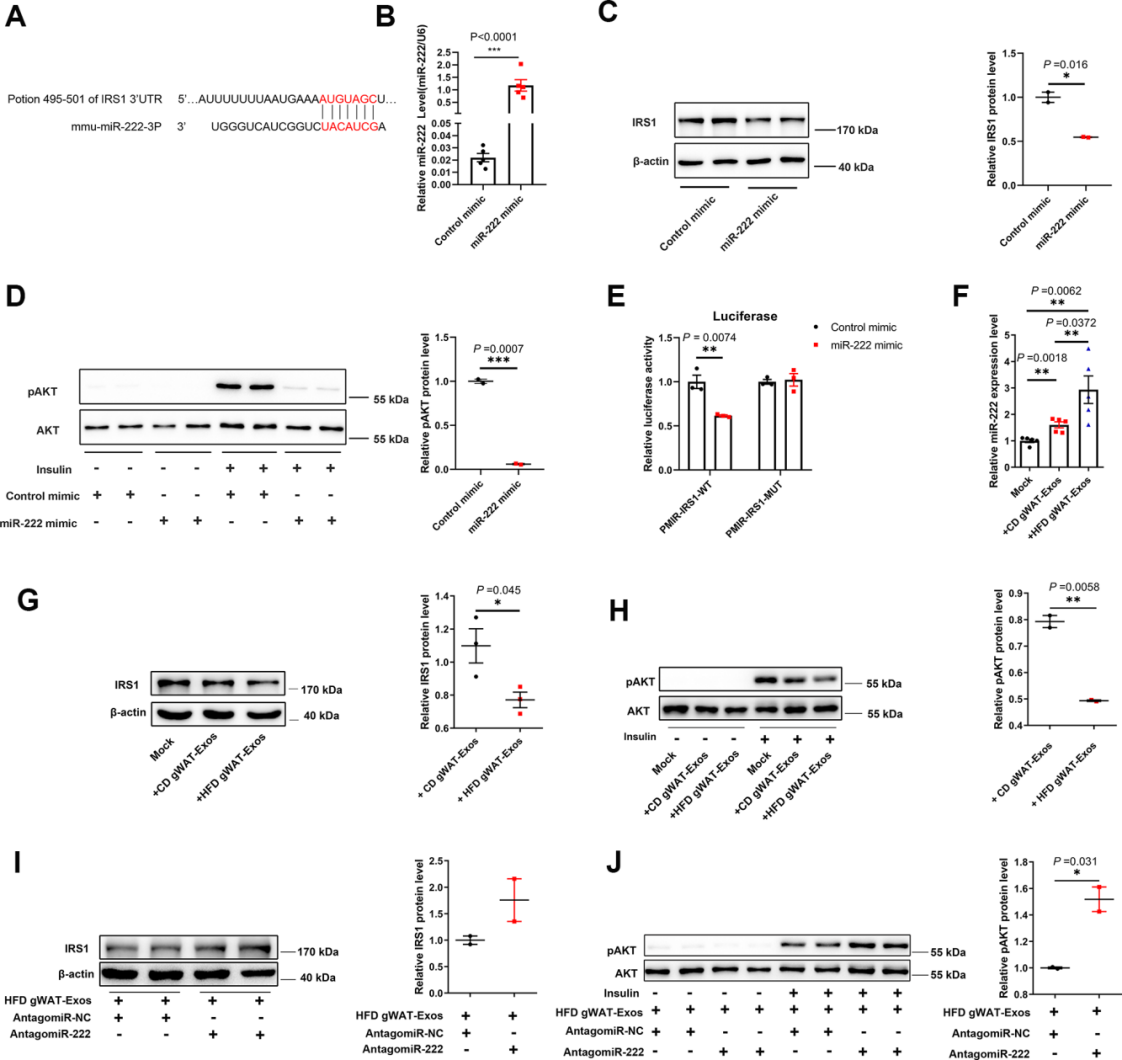
**Exosomal miR-222 secreted by gWAT inhibits the sensitivity of the insulin signal pathway by repressing IRS1.** (**A**) Representative diagram shows the results of TargetScan (http://www.targetscan.org/vert_72/) analysis predicting the presence of the miR-222 target site in the 3'-UTR of IRS-1. (**B**) QRT-PCR analysis shows the relative expression of miR-222 in the miR-NC- and miR-222 mimic-transfected Hepa 1-6 cells for 48 h (n=5 per group). (**C**) Western blot analysis shows IRS1 protein levels in the Hepa 1-6 cells transfected with miR-NC- and miR-222 mimic for 48 h. β-actin was used as loading control. (**D**) Western blot analysis shows phospho-AKT and AKT protein levels in the Hepa 1-6 cells transfected with miR-NC- and miR-222 mimic for 48 h and further treated with (+) or without (-)100 nM insulin for 10 min. The phospho-AKT levels were normalized to the total AKT levels. (**E**) Luciferase reporter activity assay results show the normalized luciferase activity in the HEK-293T cells co-transfected with miR-NC or miR-222 mimic plus pMIR-IRS1-WT plasmid or pMIR-IRS1-MUT plasmid (n=3 per group). (**F**) QRT-PCR analysis shows the relative expression of miR-222 in the Hepa 1-6 cells co-cultured with CD-gWAT-Exos or HFD-gWAT-Exos for 48h (n=5 per group). (**G**) Western blot analysis shows IRS1 protein levels in the Hepa 1-6 cells co-cultured with CD-gWAT-Exos or HFD-gWAT-Exos relative to the mock control at 48h. β-actin was used as loading control. The analysis is based on Figure 5G and Supplementary Figure 7A, 7B. (**H**) Western blot analysis shows phospho-AKT and AKT levels in the Hepa 1-6 cells co-cultured with CD-gWAT-Exos or HFD-gWAT-Exos relative to the mock control at 48h. The phospho-AKT levels were normalized to the total AKT levels. The analysis is based on Figure 5H and Supplementary Figure 7C. (**I**) Western blot analysis shows IRS1 protein levels in the antagomiR-NC- and antagomiR-222-transfected Hepa 1-6 cells co-cultured with HFD-gWAT-Exos for 48 h. β-actin was used as loading control. (**J**) Western blot analysis shows phospho-AKT and AKT levels in the antagomiR-NC- and antagomiR-222-transfected Hepa 1-6 cells co-cultured with HFD-gWAT-Exos for 48 h. The phospho-AKT levels were normalized to the total AKT levels. Note: CD-gWAT-Exos: exosomes secreted by the gWAT of CD mice; HFD-gWAT-Exos: exosomes secreted by the gWAT of HFD mice; The data are presented as the means ± SE; * *P* < 0.05, ** *P* < 0.01, *** *P* < 0.001.

Next, we investigated the target genes of miR-222 to identify the mechanism involved in obesity-induced insulin resistance. Ono et al. demonstrated that miR-222 binds to the 3’UTR of insulin receptor substrate 1 (IRS1) protein in the hepatocytes [36]. IRS1 is an important regulator of insulin signaling and the loss of IRS1 leads to insulin resistance [37–39]. Targetscan analysis suggested that IRS1 was a potential target of miR-222 (Figure 5A). Moreover, overexpression of miR-222 in the murine hepatoma cell line, Hepa 1-6 significantly reduced IRS1 protein levels compared to the controls (Figure 5B, 5C). Furthermore, insulin-induced AKT phosphorylation was significantly reduced in the miR-222-overexpressing Hepa1-6 cells compared to the controls (Figure 5D). The luciferase reporter assay showed that normalized luciferase activity was significantly reduced in the HEK-293T cells co-transfected with the luciferase reporter plasmid containing wild type 3’UTR of IRS1 (pMIR-IRS1-WT) and the miR-222 mimics compared to cells co-transfected with pMIR-IRS1-WT plasmid and the control mimic (Figure 5E). Whereas miR-222 overexpression no longer affected the normalized luciferase activity of mutated plasmid (pMIR-IRS1-MUT) since we mutated the miR-222 binding site in the IRS1 3’UTR fragment (Figure 5E). These results demonstrate that miR-222 suppresses insulin sensitivity by directly inhibiting IRS1 protein expression.

### Exosomal miR-222 secreted by the gWAT impairs insulin sensitivity by repressing IRS1

We then investigated if the exosomal miR-222 that inhibits IRS1 expression in the Hepa 1-6 cells was derived from adipose tissues. Towards this, we co-cultured Hepa 1-6 cells with CD-gWAT-Exos or HFD-gWAT-Exos and analyzed the Hepa 1-6 cells. QRT-PCR results showed that miR-222 levels were significantly higher in the Hepa 1-6 cells co-cultured with HFD-gWAT-Exos compared to those co-cultured with CD-gWAT-Exos (Figure 5F). Furthermore, IRS1 protein levels and insulin-induced phospho-AKT levels were significantly reduced in the Hepa 1-6 cells co-cultured with HFD-gWAT-Exos compared to those co-cultured with CD-gWAT-Exos (Figure 5G, 5H and Supplementary Figure 7). To further verify, we co-cultured antagomiR-NC- or antagomiR-222-transfected Hepa 1-6 cells with HFD-gWAT-Exos. The IRS1 and phospho-AKT protein levels were significantly higher in the antagomiR-222-transfected Hepa 1-6 cells co-cultured with HFD-gWAT-Exos compared to the antagomiR-NC-transfected Hepa 1-6 cells co-cultured with HFD-gWAT-Exos (Figure 5I–5J). These results confirm that the gWAT-derived exosomal miR-222 promotes insulin resistance by suppressing IRS1 protein expression.

### The gWAT-derived exosomal miR-222 represses IRS1 protein expression in the liver and skeletal muscle tissues of HFD-fed obese model mice

**Figure 6:**
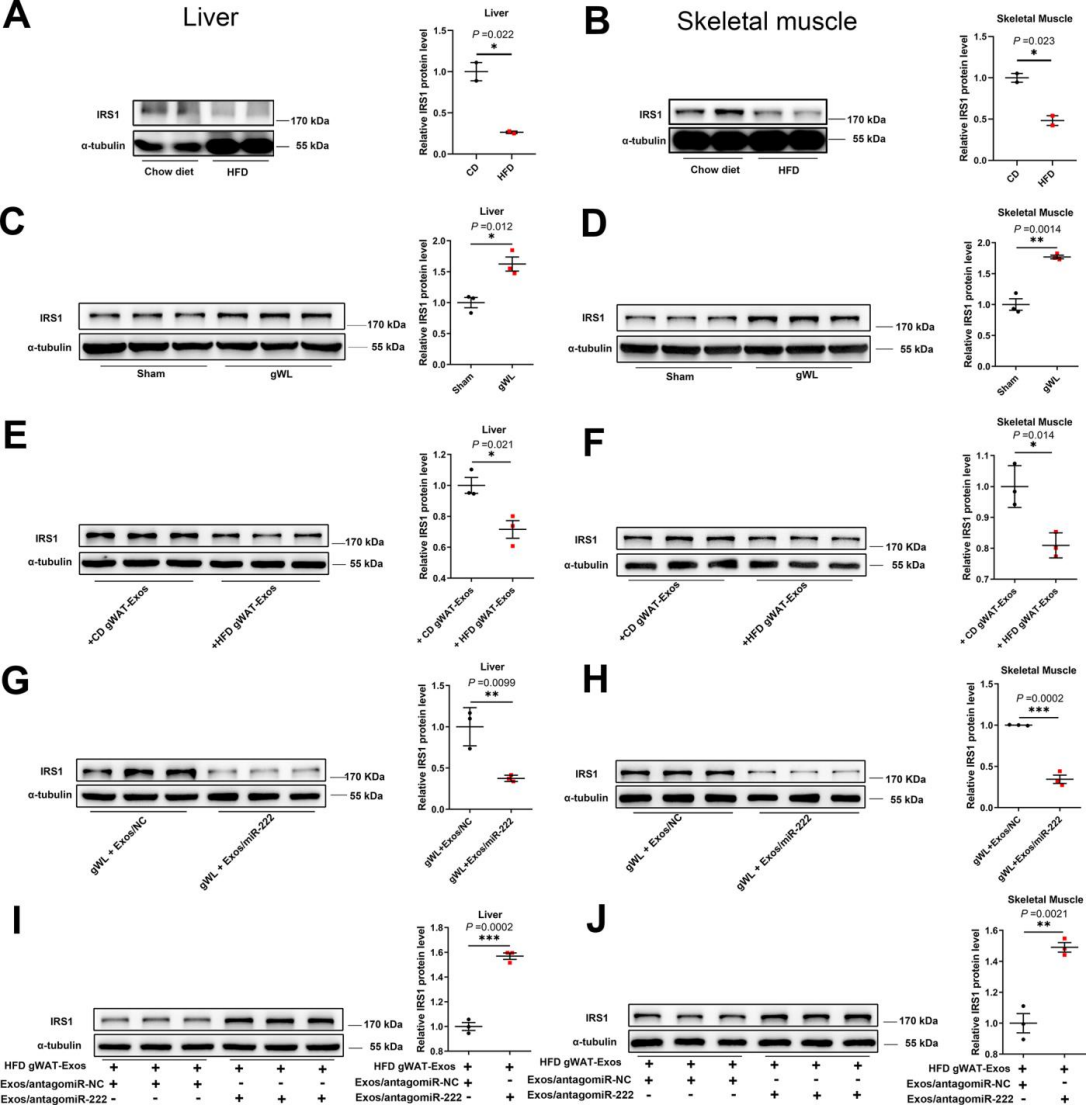
**The gWAT-derived exosomal miR-222 repress the expression of IRS1 protein in the liver and skeletal muscle tissues of HFD-fed obese model mice.** (**A**, **B**) Western blot analysis shows the IRS1 protein levels in the (**A**) liver and (**B**) skeletal muscle tissues from the CD-fed and HFD-fed male mice. (**C**, **D**) Western blot analysis shows the IRS-1 protein levels in the (**C**) liver and (**D**) skeletal muscle tissues from the gWAT-lipectomized (gWL) and sham-operated (Sham) HFD mice. (**E**, **F**) Western blot analysis shows the IRS1 protein levels in the (**E**) livers and (**F**) skeletal muscle tissues in the 8-week old wild-type mice injected continuously for 7 days via the tail vein with CD-gWAT-Exos or HFD-gWAT-Exos. (**G**, **H**) Western blot analysis shows the IRS1 levels in the (**G**) liver and (**H**) skeletal muscle tissues in the HFD gWL mice injected continuously via the tail vein for 7 days with 293T-exosomes containing miR-NC or miR-222 mimics. (**I**, **J**) Western blot analysis shows the IRS1 levels in the (**I**) liver and (**J**) skeletal muscle tissues of the wild-type mice continuously injected for 7 days with the HFD-gWAT-Exos plus 293T-exosomes (containing antagomiR-222 or antagomiR-NC). Note: CD-gWAT-Exos: exosomes secreted by the gWAT of CD mice; HFD-gWAT-Exos: exosomes secreted by the gWAT of HFD mice; The IRS1 protein levels were normalized to α-tubulin levels; The data are presented as the means ± SE; * *P* < 0.05, ** *P* < 0.01, *** *P* < 0.001.

We then analyzed the IRS1 protein levels in the liver and skeletal muscle of HFD- and CD-fed mice. The IRS1 protein levels were significantly reduced in the liver and skeletal muscle tissues of the HFD-fed obese male mice compared to those from the CD-fed male mice (Figure 6A, 6B), and similar trends were also observed in HFD-fed female mice (Supplementary Figure 8A, 8B). However, IRS1 protein levels were significantly higher in the liver and skeletal muscle tissues of the gWAT-lipectomized obese mice compared to the Sham-operated obese mice (Figure 6C, 6D). These results demonstrate that gWAT negatively regulates IRS1 protein levels in the liver and skeletal muscle tissues of HFD-fed mice.

We then investigated if gWAT-derived exosomal miR-222 negatively regulates IRS1 levels in the HFD-fed mice. Therefore, we injected gWAT-derived exosomes from the HFD-fed mice (HFD gWAT-Exos) or CD-fed mice (CD gWAT-Exos) into the 8-week old wild-type mice via the tail vein for 7 days and then analyzed IRS1 levels in the liver and skeletal muscle tissues. The IRS1 protein levels were significantly reduced in the liver and skeletal muscle tissues of mice injected with HFD gWAT-Exos compared to those injected with CD gWAT-Exos (Figure 6E, 6F). Next, we injected exosomes containing miR-222 mimic or control (miR-NC) (purified from culture medium of 293T cells) into the gWAT-lipectomized mice. The IRS1 levels were significantly reduced in the liver and skeletal muscle tissues of gWAT-lipectomized mice injected with exosomes containing miR-222 compared to those injected with exosomes containing miR-NC (Figure 6G, 6H). Moreover, IRS1 levels in the liver and skeletal muscle tissues were significantly higher in the wild-type mice that were injected with the HFD gWAT-Exos simultaneously injected with 293T-exosomes containing antagomiR-222 compared to those injected with the HFD gWAT-Exos simultaneously injected with 293T-exosomes containing antagomiR-NC (Figure 6I, 6J). Altogether, these results confirm that the gWAT-derived exosomal miR-222 suppresses IRS1 protein levels in the liver and skeletal muscle tissues in the HFD-fed obese model mice.

### The gWAT-derived exosomal miR-222 impairs insulin signaling in the liver and skeletal muscle tissues of HFD-fed obese model mice

**Figure 7:**
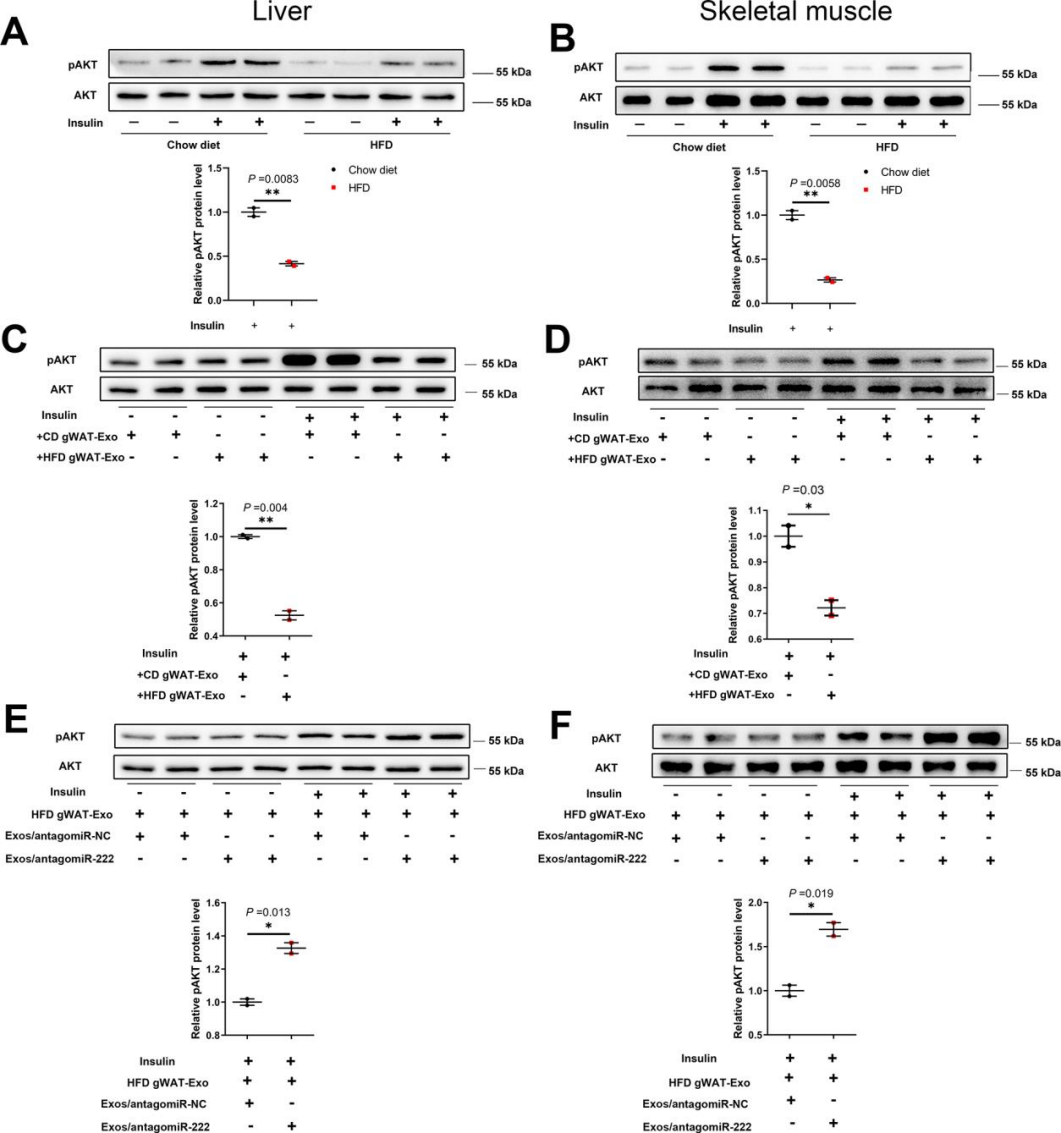
**The gWAT-derived exosomal miR-222 impairs insulin signaling in the liver and skeletal muscle tissues of HFD-fed obese model mice.** (**A**, **B**) Western blot analysis shows the phospho-AKT and AKT protein levels in the (**A**) liver and (**B**) skeletal muscle tissues from the CD-fed and HFD-fed mice. (**C**, **D**) Western blot analysis shows the phospho-AKT and AKT protein levels in the (**C**) liver and (**D**) skeletal muscle tissues in 8-week old wild-type mice continuously injected via the tail vein for 7 days with exosomes secreted by the adipose tissues from CD-fed or HFD-fed mice. (**E**, **F**) Western blot analysis shows the phospho-AKT and AKT levels in the (**E**) liver and (**F**) skeletal muscle tissues of mice continuously injected for 7 days via the tail vein with HFD-gWAT-derived exosomes plus 293T exosomes (containing antagomiR-NC or antagomiR-222). Note: For the *in vivo* insulin-stimulated AKT phosphorylation assay, the mice were injected with 0.75 IU/kg body weight insulin (i.p.) and sacrificed after 15 min. The phospho-AKT levels were normalized to the total AKT levels. The data are presented as the means ± SE; * *P* < 0.05, ** *P* < 0.01, *** *P* < 0.001.

**Figure 8:**
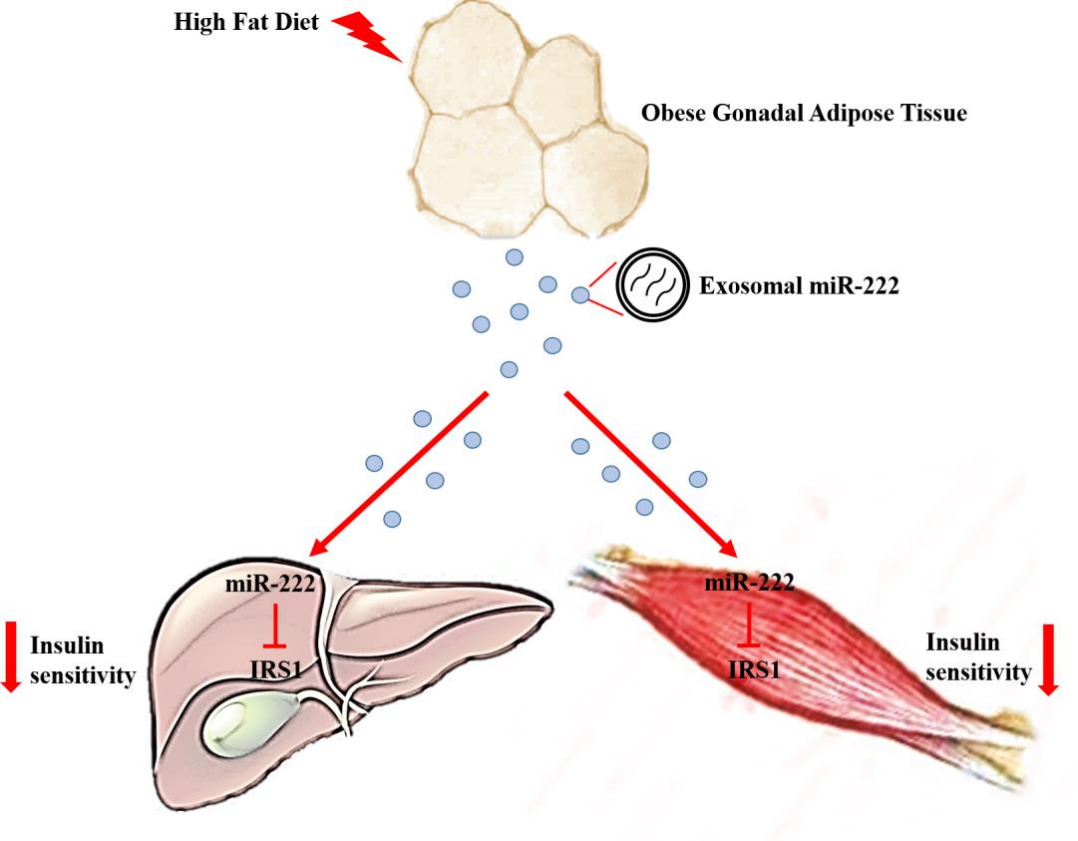
**Excessive secretion of gWAT-derived exosomal miR-222 promotes obesity-induced insulin resistance.** Upregulation of circulating gWAT-derived exosomal miR-222 promote insulin resistance by repressing IRS-1 expression in the liver and skeletal muscle tissues of the HFD-fed obese model mice.

Next, we analyzed the levels of phosphorylated AKT (phospho-AKT or pAKT) in the liver and skeletal muscle tissues of insulin-induced HFD- and CD-fed mice using western blotting. The pAKT levels were significantly decreased in the liver and skeletal muscle tissues of insulin-induced HFD-fed male mice compared to the liver and skeletal muscle tissues in the insulin-induced CD-fed male mice (Figure 7A, 7B), and similar trends were also observed in HFD-fed female mice (Supplementary Figure 8C, 8D). Furthermore, pAKT levels were significantly reduced in the liver and skeletal muscle of wild-type mice injected with the HFD gWAT-Exos compared to the liver and skeletal muscle of wild-type mice injected with the CD gWAT-Exos (Figure 7C, 7D). Moreover, pAKT levels in the liver and skeletal muscle tissues were significantly higher in the wild-type mice that were injected with the HFD gWAT-Exos simultaneously injected with 293T-exosomes containing antagomiR-222 compared to those injected with the HFD gWAT-Exos simultaneously injected with 293T-exosomes containing antagomiR-NC (Figure 7E, 7F). These results further confirm that gWAT-derived exosomal miR-222 promotes insulin resistance in the liver and skeletal muscle tissues of the obese model mice by inhibiting the insulin signaling pathway via suppression of IRS1 protein expression (Figure 8).

## DISCUSSION

Obesity is a risk factor for the development of type 2 diabetes mellitus that occurs more frequently in individuals showing insulin resistance. In this study, we demonstrate that exosomal miR-222 secreted by the gonadal white adipose tissue (gWAT) promotes obesity-induced insulin resistance.

We first observed that serum miR-222 levels were significantly higher in the T2DM patients compared to the healthy subjects. This finding was consistent with the study of Ortega et al., which reported higher circulating miR-222 levels in T2DM patients [40]. Moreover, the circulating levels of miR-222 are significantly elevated in morbidly obese patients [41]. Increased rates of T2DM have been observed among morbidly obese subjects [42]. As is well-known, insulin resistance is the hallmark of T2DM. Some of the underlying causes of insulin resistance may be overrepresented in obesity, and miR-222 may be one of these factors. Obesity is characterized by excess fat and fat is an important source of circulating miRNAs [10, 14]. Furthermore, the circulating miR-222 levels are positively associated with BMI and fat mass [41]. This suggests that adipose tissue contributes to circulating miR-222 levels. We showed that miR-222 levels are significantly increased in the white adipose tissues (WAT) in obese patients with insulin resistance compared to the healthy individuals. This suggests that the adipose tissue may contribute to the development of insulin resistance in obese individuals by secreting excessive miR-222. A previous study demonstrated that miR-222 expression in the adipose tissues correlates with the blood glucose levels in a strain-specific manner in three inbred rat strains with different diabetes susceptibility phenotypes [43]. This suggests that overexpression of miR-222 in the adipose tissues is involved in the pathophysiology of insulin resistance. Herrera et al. showed that miR-222 levels are significantly increased in cultured 3T3L1-adipocytes cultured in medium containing higher concentrations of glucose [43]. Furthermore, metformin treatment, which had a hypoglycaemic effect, had a negative effect on the circulating concentrations of miR-222 [40]. This suggests that increased serum miR-222 levels are part of the response from adipose tissues to hyperglycemic conditions. Moreover, intralipid infusion also increases circulating levels of miR-222 [40]. These findings indicated that miR-222 might be an important factor contributing to obesity induced insulin resistance.

We demonstrate that majority of the serum miR-222 is present in the exosomes. Moreover, the analysis of the GitHub data shows that adipose tissue is an important source of circulating exosomal miR-222. We analyzed different kinds of adipose tissue and found that miR-222 expression is significantly higher in the gonadal white adipose tissue (gWAT) compared to brown adipose tissue (BAT) in the wild-type and obese model mice. In the HFD-fed obese model mice, the miR-222 levels in the gWAT were almost 3-fold higher compared to those in the iWAT and BAT. Furthermore, the levels of miR-222 expression are significantly higher in the gWAT of HFD-fed obese mice compared to the wild-type mice. Furthermore, the analysis of the GitHub data shows that the transplantation of wild-type mice gWAT re-increases the reduced levels of circulating exosomal miR-222 in the ADicerKO mice. These data confirm that gWAT is a major source for the circulating exosomal miR-222 levels.

Gender-related differences in metabolic parameters have previously been reported in the metabolic syndrome studies [44, 45]. However, most studies include either males or females. In this study, we used both male and female mice to develop the HFD-fed obesity model. The high-fat diet-induced obese mice are commonly used as a model of insulin resistance. As noted in previous studies, the male mice were more severely affected by the high-fat diet (HFD) than the female mice [46–48]. In our study, we found that both the male and female HFD-fed mice exhibit insulin resistance and impaired glucose tolerance, but the impairments are worse in the male mice compared to the female mice. Moreover, serum and serum exosomal miR-222 levels were significantly higher in the HFD-induced obese mice. Both male and female HFD-fed mice showed similar trends of high circulating miR-222 levels despite gender-related changes. Therefore, we selected male mice for further experiments.

We then analyzed pri-miR-222 levels in various tissues of the HFD-fed and CD-fed mice. The pri-miR-222 levels were 8-fold higher in the gWAT of the HFD-fed obese mice compared to CD-fed mice, whereas its levels were similar in other tissues. This suggested that the source of elevated serum exosomal miR-222 in the obese mice might be gWAT. We further confirmed this by performing *in vitro* and *in vivo* experiments. We obtained gonadal adipose tissues from CD and HFD mice and cultured them *in vitro*. MiR-222 levels were higher in the HFD gWAT tissue slices as well as in the exosomes isolated from the culture medium compared to the CD gWAT tissue slices or CD gWAT exosomes. Furthermore, surgical removal of gWAT from the obese mice significantly reduced the miR-222 levels in the serum and serum exosomes. These results confirmed that gWAT secretes excessive exosomal miR-222 (gWAT exosomal miR-222) into circulation in the HFD-induced obese model mice.

We also investigated the target tissues of gWAT-exosomal miR-222 by injecting an adeno-associated virus (AAV) containing CD63-EGFP under FABP4 promoter into gWAT in situ. Confocal microscopy revealed EGFP signal in the liver, spleen, kidney and skeletal muscle tissues. The EGFP signal intensity was significantly stronger in the liver and skeletal muscle tissues. This suggests that more gWAT-derived exosomes traffic to the liver and skeletal muscle tissues. We also observed significantly elevated miR-222 levels in the liver and skeletal muscle tissues of the obese model mice. Since pri-miR-222 levels in the liver and skeletal muscle tissues were similar in both the HFD-fed and CD-fed mice, we hypothesized that gWAT-derived miR-222 may be the source of the increased miR-222 levels. We analyzed this hypothesis using gWAT-lipectomized (gWL) mice and found that miR-222 levels decreased in the liver and skeletal muscle tissues of the gWL obese model mice. This confirms that gWAT is the source of increased miR-222 levels in the liver and skeletal muscle tissues of the obese model mice.

Our previous analysis informed us that the increase of miR-222 was related to the development of insulin resistance. Therefore, we investigated the mechanisms through which miR-222 regulates insulin resistance. The miR-222-overexpressing Hepa 1-6 cells showed reduced insulin-stimulated pAKT levels. Then, we explored the mechanisms through which miR-222 suppresses insulin signaling. The IRS1 protein plays an important role in the insulin signaling pathway and mediates key metabolic changes in response to insulin stimulation [37, 38]. IRS1 knockdown mice are insulin-resistant [39]. Targetscan analysis and luciferase reporter assay confirmed that 3’-UTR of IRS1 is a direct binding target of miR-222. A previous study by Ono et al. also demonstrated that IRS1 was a direct target of miR-222 [36]. Since transfections with of miR-222 mimics does not exactly simulate the uptake of exosomal miRNA, we co-cultured hepa 1-6 cells with purified exosomes from the culture medium of gWAT tissues isolated from the CD-fed mice (CD gWAT-Exos) and HFD-fed mice (HFD gWAT-Exos). We observed significant increase in the miR-222 levels and significant reduction of IRS1 and phospho-AKT protein levels in the Hepa1-6 cells co-cultured with HFD gWAT-Exos compared to the controls. However, HFD-gWAT-Exos co-cultured Hepa 1-6 cells transfected with antagomiR-222 showed higher IRS1 and phospho-AKT levels than that transfected with antagomiR-NC. These results suggest that miR-222 from the HFD-gWAT-exosomes suppresses insulin signaling pathway by suppressing IRS1 protein expression.

Liver and skeletal muscles are the major target tissues of gWAT-derived exosomal miR-222 in obese model mice. Therefore, we tested if the gWAT-derived exosomal miR-222 induces insulin resistance in the liver and skeletal muscle tissues of HFD-fed obese model mice by repressing IRS1. We observed significantly higher miR-222 levels and reduced IRS1 in the liver and skeletal muscle tissues of the HFD-fed obese model mice. We further observed that IRS1 were significantly increased in the liver and skeletal muscle tissues of gWAT-lipectomized (gWL) obese mice, thereby confirming the essential role of gWAT in the repression effect on IRS1. Furthermore, the IRS1 protein levels in livers and skeletal muscles of wild-type mice were decreased by the injections of HFD-gWAT-Exos, demonstrating the repressing effect of HFD-gWAT-Exos on IRS1. Moreover, we observed that the IRS1 protein levels in the livers and skeletal muscles were significantly re-decreased by the injections of 293t-exosomes containing miR-222 mimics in gWL mice and significantly re-increased by injections with 293T-exosomes containing antagomiR-222 in HFD-gWAT-Exos injected mice. These results confirm that gWAT-exosomal miR-222 supresses IRS1 protein expressions in the liver and skeletal muscle tissues of HFD-fed obese model mice.

IRS1 is an important modulator in insulin signal pathway [37, 38]. In accordance with the reduced IRS-1, we observed insulin-stimulated pAKT levels in liver and skeletal muscle tissues were significantly decreased in obese model mice. Furthermore, insulin-stimulated pAKT levels in liver and skeletal muscle tissues of wild-type mice were significantly reduced by the injections of HFD-gWAT-Exos and then re-improved by the injections of 293T-exosomes containing antagomiR-222. Altogether, these results confirm that gWAT-exosomal miR-222 inhibits the insulin signalling pathway in the liver and skeletal muscle tissues of obese mice by suppressing IRS1 protein expression.

Obesity can be thought of as a premature metabolic disorder similar to ageing, and the dysfunction of adipose in obesity may be responsible for the metabolic dysfunction linked to ageing [49]. Furthermore, it is the visceral fats especially epididymal fats that are considered to relate to lifespan. Surgical removal of visceral fat extends longevity and the improvement of insulin sensitivity may play an important role in that, which is similar to calorie restriction (CR) [50]. In our study, we found exosomal miR-222 secreted by gWAT in obese mice impaired the insulin sensitivity of livers and skeletal muscles by repressing IRS1. This mechanism may be also involved in the effect of visceral fat on lifespan. However, this aspect needs to be addressed in future studies.

In conclusion, our study demonstrates that increased levels of gWAT-derived exosomal miR-222 in the serum of obese model mice promote insulin resistance in the liver and skeletal muscle tissues by repressing IRS-1. Our findings suggest that gWAT-derived exosomal miR-222 is a potential target for treating obesity-induced metabolic syndrome and type 2 diabetes.

## MATERIALS AND METHODS

### Bioinformatics data

The RNA-seq data from the GSE90028 dataset (GPL11154 Illumina HiSeq 2000 platform) for the type-2 diabetes mellitus (T2DM) patients (n=7) and healthy subjects (n=16) was downloaded from the NCBI GEO database (http://www.ncbi.nlm.nih.gov/geo/query/acc.cgi?acc=GSE90028) and is shown in Supplementary Table 1.

The miRNA expression profiles from the GSE25402 dataset, which is based on the GPL8786 Affymetrix Multispecies miRNA-1 Array platform was downloaded from NCBI GEO (https://www.ncbi.nlm.nih.gov/geo/query/acc.cgi?acc=GSE25402) database. The study subjects were subdivided based on age and obesity into four groups: young lean (n=11), young obese (n=13), old lean (n=14) and old obese (n=16) as shown in Supplementary Table 2.

We downloaded the microRNA data for the wild-type and adipose-tissue specific knockout mice (fat-exosome-microrna/Ct_tables/ADicerKO-6mo-Ct.csv and fat-exosome-microrna/Ct_tables/transplant-exo-Ct.csv) from the GitHub (https://github.com/jdreyf/fat-exosome-microrna) database. The RNA expression profiling arrays from the GSE81976 dataset that was generated using the GPL7723 miRCURY LNA microRNA Array v.11.0 platform were downloaded from the NCBI GEO database (https://www.ncbi.nlm.nih.gov/geo/query/acc.cgi?acc=GSE81976). The RNA expression profiling arrays from the GSE85101 dataset that were generated using the GPL9250 Illumina Genome Analyzer II (*Mus musculus*) platform were downloaded from the NCBI GEO database (https://www.ncbi.nlm.nih.gov/geo/query/acc.cgi?acc=GSE85101). All the data was processed and analyzed using the GraphPad Prism 6 statistical software and the IDEP 9.0 software (http://bioinformatics.sdstate.edu/idep/).

### Animal studies

All animal procedures were performed in accordance with the guidelines of the Institutional Animal Care and Use Committee. We purchased 8-week old C57BL/6 male and female mice from the Model Animal Research Center of Nanjing University and housed them in a temperature-controlled animal facility under a 12/12 hour light-dark cycle. Eight-week old male and female mice were fed a normal chow diet (CD) or a high-fat diet (HFD; 60% kcal% fat; D12492, Research Diets, NJ, USA) for 8 weeks. The body weight and food intake was measured every 3 days for all the mice. The mice were subjected to intraperitoneal glucose tolerance test (IPGTT) and insulin tolerance test (ITT) after 8 weeks of diet.

For IPGTT, mice were intraperitoneally (i.p) injected with 1 g/kg body weight of dextrose after 12 h of fasting. The blood glucose levels were then measured at 0, 30, 60, and 120 min.

For ITT, mice underwent fasting for 6 h. Then, they were intraperitoneally injected with 0.75 IU/kg body weight insulin. The blood glucose levels were measured at 0, 15, 30, 45, and 60 min after injection by collecting blood from the tail tip.

For the *in vivo* insulin-stimulated AKT phosphorylation assay, the mice were injected with 0.75 IU/kg body weight insulin (i.p.) and sacrificed after 15 min.

We performed gonadal white adipose tissue (gWAT) lipectomy by surgically removing the major gWAT pads as previously described [49]. The testicular blood supply was not affected. For the sham group, the abdominal cavity was opened, and gWAT was mobilized but not removed.

For the AAV infection experiments, we purchased AAV viruses (HBAAV2/9-FABP4-3xflag-CD63-EGFP-T2A-mCherry) at titres of 2.0x10^12^ viral genomes / mL from the HANBIO Co. Ltd. (Shanghai, China). Then, the abdominal cavity of the mice was surgically opened and 5μl AAV was injected at multiple sites into both lateral gWAT fat pads (5ul AAV per site). For the control group, the abdominal cavity was opened, but the gWAT was not injected with AAV. At 8 weeks after injection, the mice were imaged by the IVIS Lumina XR in vivo imaging system (PerkinElmer, USA).

### Fluorescence microscopy

At 8 weeks after injection, the mice were sacrificed. Before collecting the tissues, whole body perfusion was performed on mice by first perfusing PBS and then fixing the tissues by perfusing the body with 4% PFA. The tissues (including heart, liver, spleen, lung, kidney, brain, skeletal muscle, gWAT) were harvested, fixed in 4% PFA, and dehydrated with 30% sucrose solution. Tissue samples were embedded in O.C.T. (opti-mum cutting temperature compound, 4583, SAKURA, USA) and frozen in -80°C. The adipose tissues were then cut into 30-μm-thick sections and the other tissues were cut into 12-μm-thick sections. The nuclei were stained with DAPI. The sections were imaged using the confocal microscope (TCS SP8 CARS, Leica, Germany).

### Cell culture

The cell lines hepa 1-6 and HEK-293T were purchased from the Shanghai Institute of Biochemistry and Cell Biology, Chinese Academy of Sciences (Shanghai, China). The cells were cultured in the Dulbecco’s modified Eagle’s medium (DMEM) (Gibco, Carlsbad, CA, USA) containing 10% fetal bovine serum (FBS; Gibco) and 1% penicillin–streptomycin (Gibco) in the humidified incubator at 5% CO_2_ and 37 °C.

### Cell transfections

Synthetic miR-222 mimic and inhibitor and negative control oligos were purchased from GenePharma (Shanghai, China). The Cells were transfected using Lipofectamine 2000 (Invitrogen, CA, USA). The concentration of miRNA we used was according to the manufacturer’s protocol of lipofectamine2000. At 6h after transfection, the cell culture medium was replaced by fresh DMEM medium containing 10% FBS for a further 48 h. For hepa 1-6 cells, the cells were harvested for RNA isolation and protein extraction. Transfection efficiency was assessed using qRT-PCR. For 293T cells, the culture medium was collected for exosomes isolation (FBS added in this medium was exosomes-depleted).

### Luciferase reporter assay

A 582-bp fragment of the wild-type 3’UTR containing the potential miR-222 binding site were inserted into a luciferase reporter vector (pMIR-REPORT Luciferase miRNA Expression Reporter Vector, AM5795, Applied Biosystems, Foster City, CA). This clone was called pMIR-IRS1-WT. To verify the binding specificity, we mutated the seed region for miR-222 from ATGTAGC to TACATCG. The synthetic mutant IRS1 3’UTR fragment was inserted into an equivalent luciferase reporter plasmid. This clone was called pMIR-IRS1-MUT. These plasmids were purchased from GenScript Co. Ltd. (Nanjing, China). For the luciferase reporter assay, the 293T cells were cultured in 24-well plates, and co-transfected with 50pmol miR-222 mimics (or miR-NC), 0.2ug luciferase plasmid (pMIR-IRS1-WT or pMIR-IRS1-MUT) and 0.2ug β-Gal plasmid (pMIR-REPORT β-gal Control Plasmid, Applied Biosystems) using Lipofectamine 2000 (Invitrogen). The β-Gal plasmid was used as a control reporter for normalization. After 48 h, the luciferase activity was measured using the Luciferase Assay System kit (E1501, Promega, Madison, WI, USA) according to manufacturer’s protocol.

### Quantitative real-time PCR

Total RNA was extracted from cells and tissues using TRIzol (15596-018, Invitrogen, Carlsbad, CA, USA) according to the manufacturer’s protocol. Total RNA from the serum samples was extracted using TRIzol LS (10296-028, Invitrogen) according to the manufacturer’s protocol. Total RNA from the exosomes was isolated using the miRCURY Biofluid RNA isolation kit (EXIQON, Aarhus, Denmark). To quantify mature miR-222, TaqMan miRNA Assay Probes (Assay ID: 002276, Applied Biosystems, Foster City, CA) were used according to the manufacturer’s instructions. qPCR was performed using a LightCycler 480 II system (Roche, Mannheim, Germany). For cellular miRNA expression, the miR-222 levels were normalized to the U6 levels detected using the U6-specific Taqman probe (Assay ID: 001093, Applied Biosystems). For exosomal mi-222 expression, the data was estimated using a standard curve. The pri-miR-222 levels were detected using the pri-miR-222-specific TaqMan probes (Assay ID: Mm03307187, Applied Biosystems) and the data was normalized to the GAPDH levels. The relative levels were estimated using the 2^−ΔΔCt^ ^(ΔCt sample – ΔCt control)^ method. GAPDH (sense): GGTGAAGGTCGGTGTGAACG; and GAPDH (antisense): CTCGCTCCTGGAAGATGGTG.

### Western blotting

Total protein lysates from cells and tissues were extracted in RIPA buffer (P0013B, Beyotime, Shanghai, China) containing protease and phosphatase inhibitors. The protein concentrations were measured using the BCA protein assay kit (23225, Thermo Fisher Scientific, Rockford, Illinois, USA). Equal amounts of protein were separated on a 10% SDS-PAGE and transferred onto the polyvinylidene difluoride (PVDF) membranes (Roche, Mannheim, Germany). The membranes were then blocked with 5% non-fat powdered milk (A600669, Sangon, Shanghai, China) in TBST (10 mM Tris–HCl, 150 mM NaCl and 0.1% (v/v) Tween-20) for 1 h at room temperature. Then, the membranes were incubated overnight at 4 °C with primary antibodies against IRS-1 (ab131487, Abcam, Cambridge, UK), p-AKT (4060s, CST, MA, USA), AKT (9272s, CST) β-actin (8457s, CST), and α-tubulin (sc-5286, Santa Cruz, CA, USA). The membranes were washed 6 times with TBST for 5 mins each and then incubated with the HRP-conjugated goat anti-rabbit antibodies (7074s, CST) and HRP-conjugated goat anti-mouse antibodies (7076s, CST) for 1 h at room temperature. The protein bands were then developed using the SuperSignal West Pico Chemiluminescent Substrate (34580, Thermo Fisher Scientific) and visualized with the Tanon imaging system (5200S, Tanon, China). The protein bands were analyzed using the Image J software. The p-AKT protein levels were normalized to the total AKT levels, whereas the IRS-1 protein levels were normalized to the β-actin or α-tubulin levels.

### Isolation of exosomes

### Isolation of serum exosomes

The mouse serum was centrifuged at 1000 x g for 5 min and then the supernatant was centrifuged at 10000 x g for 30 min to remove the cell debris. The supernatants containing the clarified serum were combined with 0.2 volumes of the Total Exosome Isolation (from serum) reagent (4478360, Thermo Fisher Scientific). Then, the exosomes were harvested according to the manufacturer’s protocol. The exosomes were resuspended in PBS.

### Isolation of 293T-Exosomes

The culture medium of 293T cells was collected, centrifuged at 1000 x g for 5 min, and then the supernatant was centrifuged at 10000 x g for 30min. The supernatant was filtered through a 0.22-μm filter and further centrifuged at 110000 x g for 70 min to obtain the exosomal pellet, which was re-suspended in PBS.

### Isolation of gWAT-Exosomes

The gWAT fat pads were surgically harvested from the CD- or HFD-fed mice, placed in a 10 cm culture dish and cut into small pieces (~ 0.5-1 mm^3^). Then, the minced gWAT pieces were cultured in DMEM medium (Gibco) containing 10% FBS (Gibco) and 1% penicillin–streptomycin (Gibco) in an humidified incubator at 5% CO_2_ and 37 °C for 48 h (FBS was exosomes-depleted). Then, the medium was centrifuged at 1000 x g for 5 min to remove the cells and then further centrifuged at 10000 x g for 30 min to remove cell debris. The supernatant was filtered through a 0.22-μm filter and further centrifuged at 110000 x g for 70 min to obtain the exosomal pellet, which was re-suspended in PBS. A pair gWAT pads from the same mouse were plated in a dish. After 48h culture, a pair gWAT pads from CD-fed mouse secreted ~30 μg (protein) exosomes and a pair gWAT pads from HFD-fed mouse secreted ~50 μg (protein) exosomes.

### Exosomes treatment

*In vitro* exosomes treatments: 10 μg (protein) exosomes were added to 1×10^5^ recipient cells.

*In vivo* exosomes treatments: For 293T exosomes, ~30 μg (protein) exosomes were injected into a mouse every time. For exosomes derived from gWAT, the exosomes derived from a pair gWAT pads (derived from CD or HFD mice) were injected into a mouse every time. For injection, we performed tail-vein-injections every day for 7 days.

### Statistical analysis

All images of western blot and immunofluorescence are representative of at least three independent experiments. The data are presented as the means ± standard error (SE). The samples were compared using the two-tailed Student’s *t*-tests. A *P* value less than 0.05 was considered statistically significant. All statistical analyses were carried out using the GraphPad Prism 8.0 software.

## Supplementary Material

Supplementary Figures

Supplementary Tables
