# Supplementary material for: Gonadal white adipose tissue-derived exosomal MiR-222 promotes obesity-associated insulin resistance
Source: Aging (Albany NY). 2020 Nov 10;12(22):22719–43. doi: 10.18632/aging.103891 (PMC7746358; doi:10.18632/aging.103891)
Supplement: Supplementary Figures [file aging-12-103891-s001..pdf]

SUPPLEMENTARY TABLES

Supplementary Table 1. The data selected from GSE90028.

	Number of subjects	Accession numbers
Control	16	GSM2395967, GSM2395968, GSM2395969, GSM2395970, GSM2395971, GSM2395972, GSM2395973, GSM2395974, GSM2395984, GSM2395985, GSM2395986, GSM2395987, GSM2395988, GSM2395989, GSM2395990, GSM2395991,
T2DM	7	GSM2395951, GSM2395952, GSM2395953, GSM2395954, GSM2395955, GSM2395956, GSM2395957,
SUM	23	

Note: Control: healthy individuals; T2DM: type 2 diabetes mellitus patients.

Supplementary Table 2. The data selected from GSE25402.

	Obesity status	Age	Number of subjects	Accession numbers
Non-obese 1	Non-obese	< 40	11	GSM625476, GSM625505, GSM625478, GSM625513, GSM625516, GSM625502, GSM625468, GSM625494, GSM625490, GSM625467, GSM625477
Obese 1	Obese	< 40	13	GSM625504, GSM625488, GSM625473, GSM625480, GSM625503, GSM625514, GSM625469, GSM625487, GSM625517, GSM625474, GSM625508, GSM625486, GSM625515
Non-obese 2	Non-obese	> 40	14	GSM625475, GSM625499, GSM625462, GSM625497, GSM625495, GSM625498, GSM625496, GSM625465, GSM625464, GSM625472, GSM625500, GSM625463, GSM625491, GSM625493
Obese 2	Obese	> 40	16	GSM625484, GSM625485, GSM625511, GSM625482, GSM625509, GSM625501, GSM625506, GSM625512, GSM625507, GSM625479, GSM625489, GSM625510, GSM625492, GSM625471, GSM625470, GSM625466
	Nonobese		25	
	Obese		29	
		<40	24	
		>40	30	
Sum			54	
