# Supplementary material for: Gonadal white adipose tissue-derived exosomal MiR-222 promotes obesity-associated insulin resistance
Source: Aging (Albany NY). 2020 Nov 10;12(22):22719–43. doi: 10.18632/aging.103891 (PMC7746358; doi:10.18632/aging.103891)
Supplement: Supplementary Tables [file aging-12-103891-s002..pdf]

SUPPLEMENTARY FIGURES

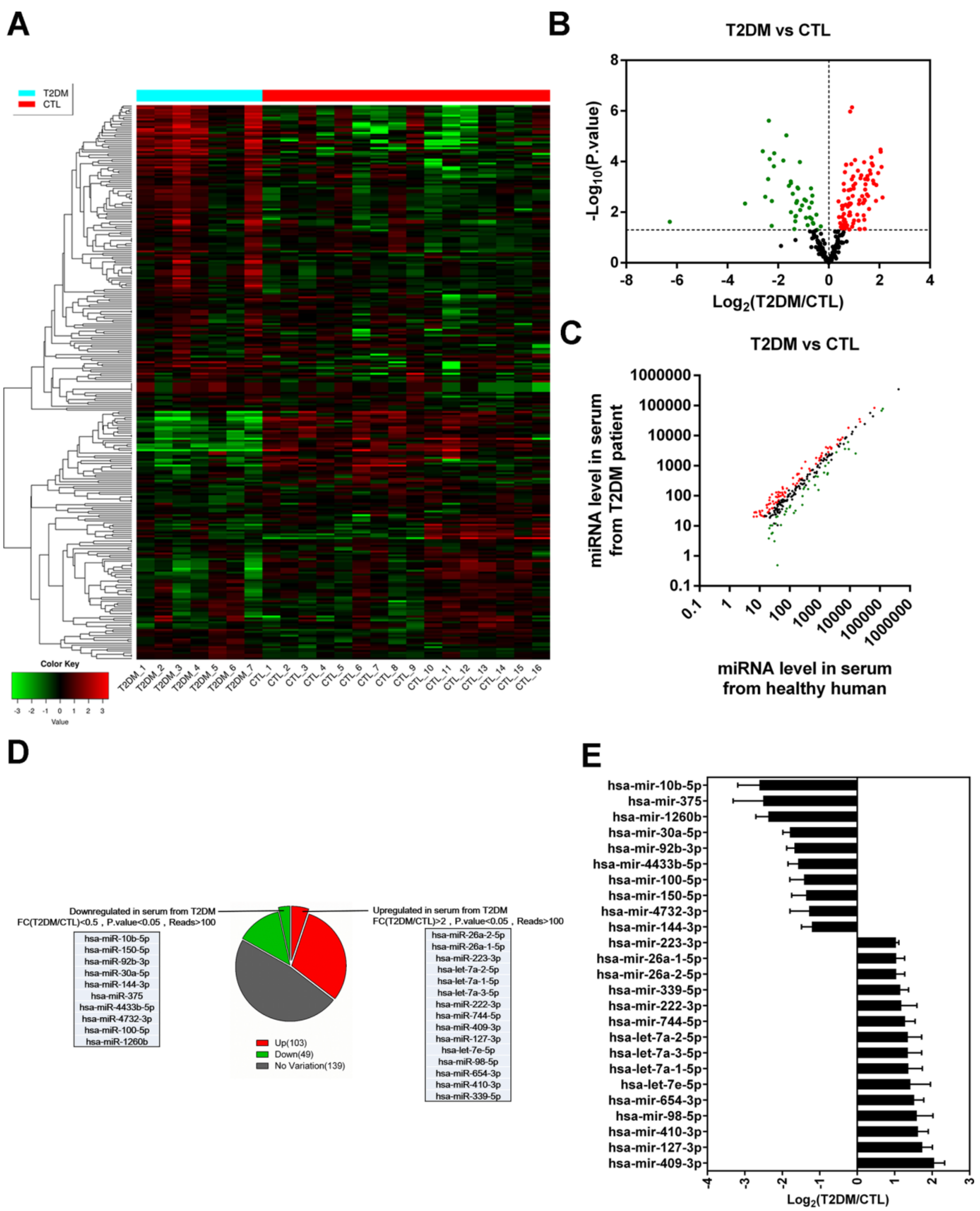

Supplementary Figure 1. The analysis of GSE90028 dataset. (**A**) Heat map shows the differential expression of the selected miRNAs with at least 20 reads in all the serum samples from T2DM patients (n=7) and healthy individuals (n=16). (**B**) The volcano plot of $\log_2$ (T2DM/CTL) on the X-axis versus $-\log_{10}$ (*P*-value) on the y-axis shows the distribution of 291 selected miRNAs. (**C**) The intensity scatter plot shows the comparison of the read counts of 291 selected miRNAs in the serum of healthy subjects (X-axis) and T2DM patients (Y-axis). (**D**) The pie chart shows the proportion. The salient parts of the pie chart represent those selected miRNAs with atleast 100 reads in all the serum samples of T2DM and healthy subjects and FC (T2DM/CTL) > 2 or < 0.5. (**B**–**D**) Based on the $P < 0.05$ as the cut-off criteria, red represent upregulated miRNAs, green represent downregulated miRNAs and black represent unaltered miRNAs in the T2DM patients. (**E**) The waterfall plot shows the fold change in the expression of the upregulated or downregulated miRNAs in the T2DM patients. The X-axis represents $\log_2$ (T2DM/CTL). The data are presented as the means ± SE; * $P < 0.05$, ** $P < 0.01$, *** $P < 0.001$.

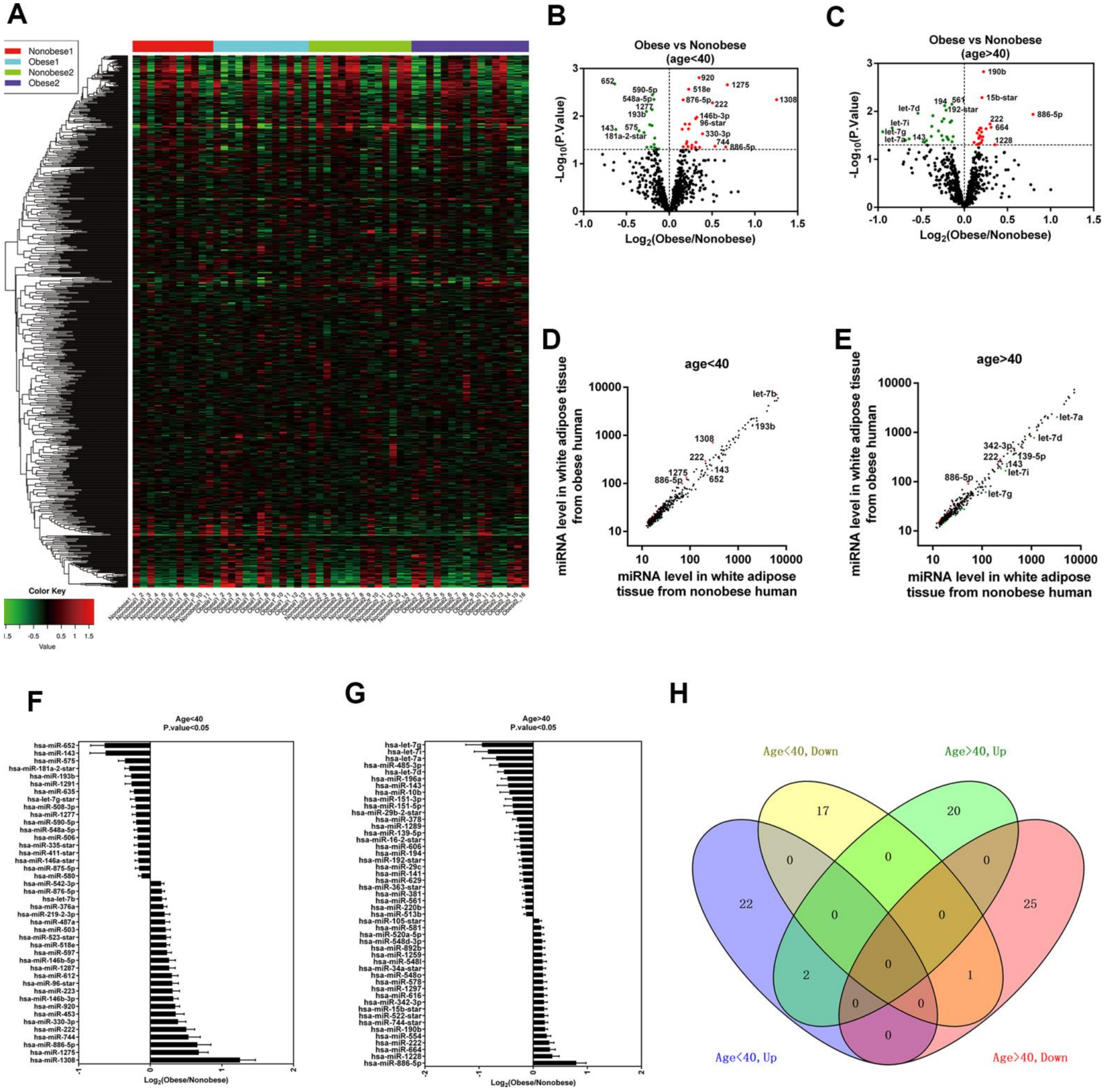

Supplementary Figure 2. The analysis of the GSE25402 dataset. (**A**) The heat map shows the differential expression of miRNAs in white adipose tissues from the young non-obese (Non-obese 1 group; n=11), young obese (Obese 1 group; n=13), old non-obese (Non-obese 2 group; n=14), and old obese (Obese 2 group, n=16) groups in the GSE25402 dataset. (**B**, **C**) The volcano plots of $\log_2$ (young obese/young non-obese) or $\log_2$ (old obese/old non-obese) on the X-axis versus $-\log_{10}$ (*P*-value) on the y-axis show the expression of the miRNAs in the white adipose tissues of the (**B**) young and (**C**) old groups. (**D**, **E**) The intensity scatter plots show the differential expression of the miRNAs in the white adipose tissues of (**D**) young obese vs. young non-obese individuals and (**E**) old obese vs. old non-obese individuals. (**B**–**E**) Based on the $P < 0.05$ as the cut-off criteria, red represent upregulated miRNAs, green represent downregulated miRNAs and black represent unaltered miRNAs in the obese patients. (**F**, **G**) The waterfall plots show the fold changes of significantly upregulated and downregulated miRNAs in the white adipose tissues of (**F**) young and (**G**) old obese patients relative to the corresponding non-obese individuals. The X-axis represents $\log_2$ (Obese/Non-obese). (**H**) The Venn diagram shows the overlap between differentially expressed (up- or down-regulated) miRNAs in the white adipose tissues of young and old obese individuals.

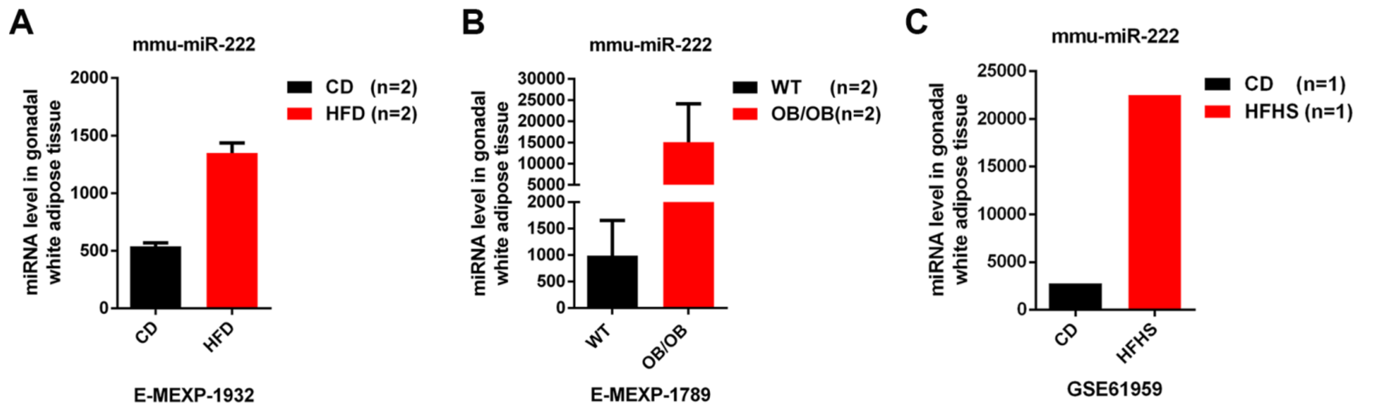

Supplementary Figure 3. MiR-222 is significantly upregulated in the gonadal adipose tissues of various diabetic mouse models. (**A**) The miR-222 levels in the gonadal adipose tissues of mice fed on a chow diet (black bar) or a high-fat diet (red bar). The data is based on the E-MEXP-1932 dataset (https://www.ebi.ac.uk/arrayexpress/). (**B**) The miR-222 levels in the gonadal adipose tissues of WT (black bar) and OB/OB (red bar) mice. The data is based on the E-MEXP-1789 dataset (https://www.ebi.ac.uk/arrayexpress/). (**C**) The miR-222 levels in the gonadal adipose tissues of mice fed on a chow diet (black bar) or a high-fat high-sugar diet (HFHS; red bar). The data is based on the GSE61959 dataset that was downloaded from the GEO database.

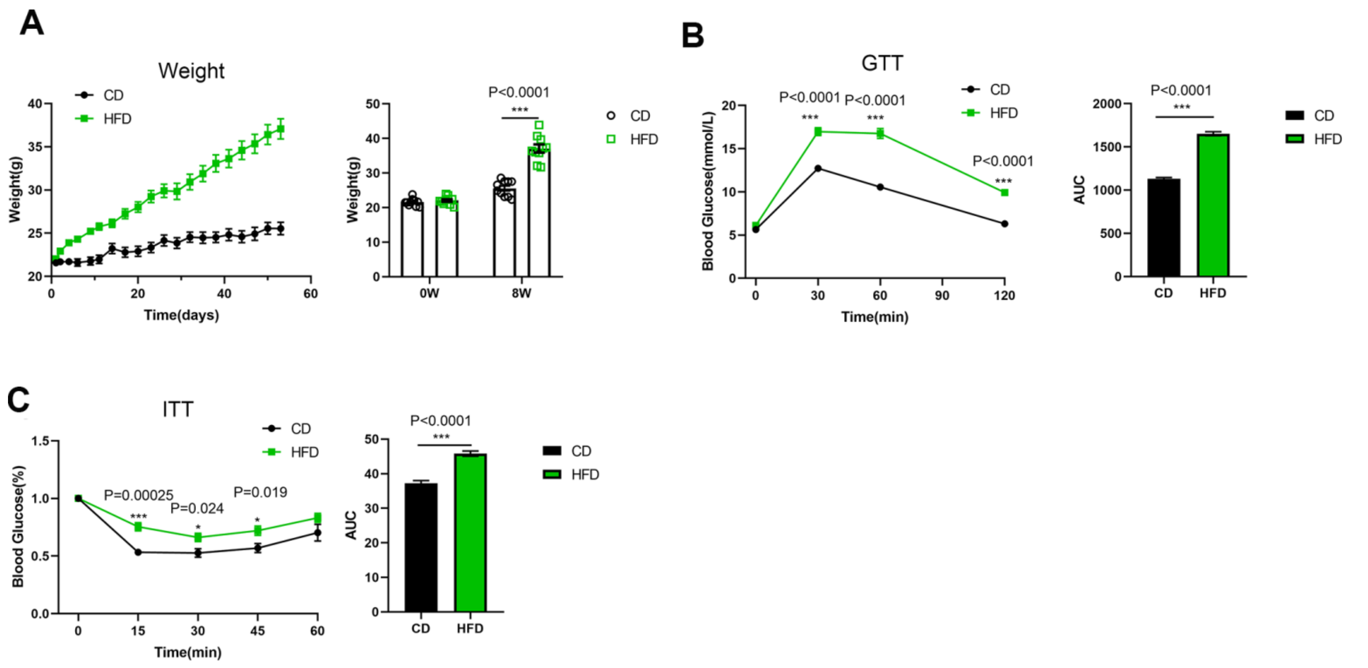

Supplementary Figure 4. The metabolic characteristics of CD- and HFD-fed male mice. (**A**) The curve shows body weight of CD-fed (black line) and HFD-fed (green line) male mice (n=10 per group) over a period of 8 weeks. The body weight was measured every 3 days for 8 weeks. (**B**) The results of the glucose tolerance test (GTT; left) and the area under the curve (AUC) of the GTT (right) is shown for the CD-fed (black line or bar) and HFD-fed (green line or bar) male mice (n=5-10 per group). The GTT was performed after 8 weeks of diet. (**C**) The results of the insulin tolerance test (ITT; left) and the area under the curve (AUC) of the ITT (right) is shown for the CD-fed (black line or bar) and HFD-fed (green line or bar) male mice (n=7-10 per group). The ITT was performed after 8 weeks of diet. . Note: CD, chow diet; HFD, high fat diet; Data are presented as means ± SE; * $P < 0.05$, **, *** $P < 0.001$.

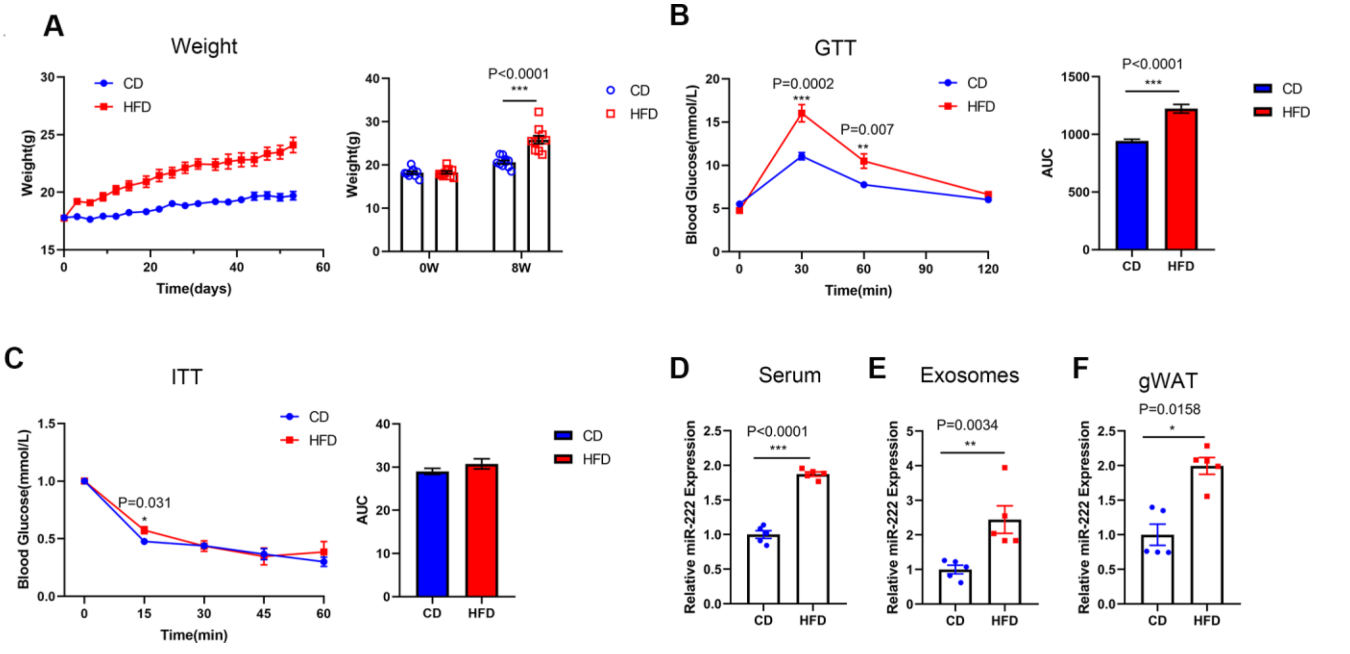

Supplementary Figure 5. The metabolic characteristics of the CD-fed and HFD-fed female mice. (**A**) The curve shows the body weight of CD-fed (blue line) and HFD-fed (red line) female mice (n=10 per group) over a period of 8 weeks. The body weight was measured every 3 days for 8 weeks. (**B**) The results of the glucose tolerance test (GTT; left) and the area under the curve (AUC) of the GTT (right) is shown for the CD-fed (blue line or bar) and HFD-fed (red line or bar) female mice (n=5-10 per group). The GTT was performed after 8 weeks of diet. (**C**) The results of the insulin tolerance test (ITT; left) and the area under the curve (AUC) of the ITT (right) is shown for the CD-fed (blue line or bar) and HFD-fed (red line or bar) female mice (n=7-10 per group). The ITT was performed after 8 weeks of diet. (**D**–**F**) The relative miR-222 expression in the (**D**) serum, (**E**) serum exosomes and (**F**) gonadal white adipose tissues from the CD-fed and HFD-fed female mice (n=5 per group) is shown. Note: CD, chow diet; HFD, high fat diet; Data are presented as means ± SE; * $P < 0.05$, ** $P < 0.01$, *** $P < 0.001$.

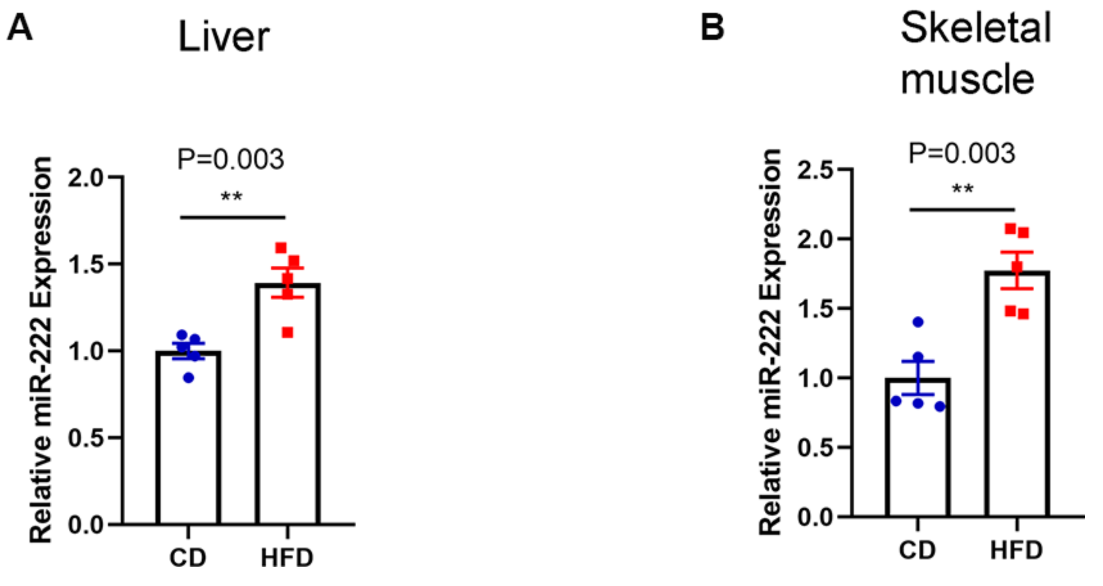

Supplementary Figure 6. The relative miR-222 expression levels in the liver and skeletal muscle tissues of HFD-fed female mice. (**A**, **B**) QRT-PCR analysis shows relative miR-222 levels in the (**A**) liver and (**B**) skeletal muscle tissues from the CD-fed and HFD-fed female mice (n=5 per group). The data are presented as the means ± SE. * $P < 0.05$, ** $P < 0.01$, *** $P < 0.001$.

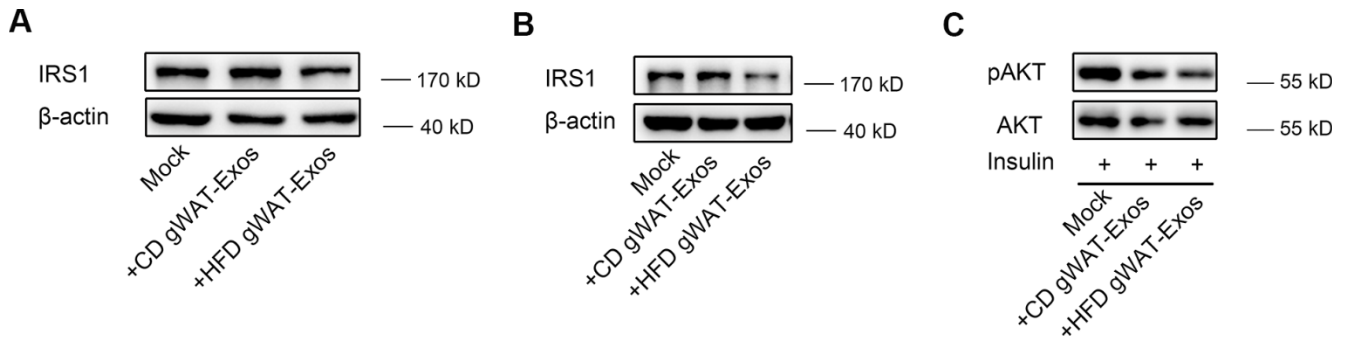

Supplementary Figure 7. Western blot analysis of IRS1 and pAKT levels in the Hepa 1-6 cells co-cultured with CD-gWAT- or HFD-gWAT-Exosomes. (**A**, **B**) Representative images show the IRS1 and β-actin protein levels in the Hepa 1-6 cells co-cultured with CD-gWAT-Exos or HFD-gWAT-Exos for 48h. Related to Figure 5G. (**C**) Representative image shows the phospho-AKT and AKT protein levels in the Hepa 1-6 cells co-cultured with CD-gWAT-Exos or HFD-gWAT-Exos for 48h. Related to Figure 5H.

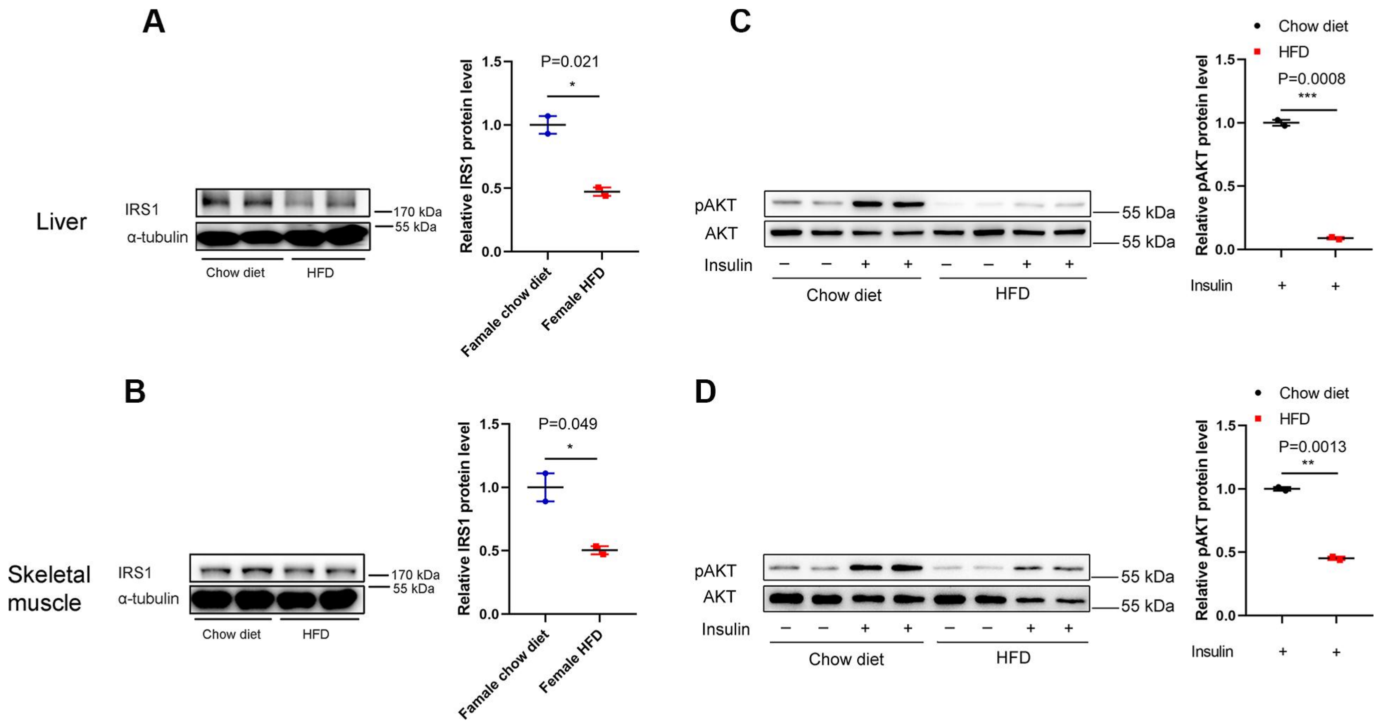

Supplementary Figure 8. Western blot analysis of IRS1 and pAKT levels in the liver and skeletal muscle tissues of HFD-fed female mice. (**A**, **B**) Western blot analysis shows IRS1 and α-tubulin expression in the (**A**) liver and (**B**) skeletal muscle tissues from the CD-fed and HFD-fed female mice. The IRS1 protein levels were normalized to α-tubulin levels. (**C**, **D**) Western blot analysis shows phospho-AKT and AKT levels in the (**C**) liver and (**D**) skeletal muscle tissues from the CD-fed and HFD-fed female mice treated with (+) or without (-) insulin. Phospho-AKT levels were normalized to the total AKT levels. The data are presented as the means ± SE. * $P < 0.05$, ** $P < 0.01$, *** $P < 0.001$.
